# The Manú Gradient as a study system for bird pollination

**DOI:** 10.3897/BDJ.6.e22241

**Published:** 2018-03-02

**Authors:** Mannfred MA Boehm, Micah N Scholer, Jeremiah JC Kennedy, Julian M Heavyside, Aniceto Daza, David Guevara-Apaza, Jill E Jankowski

**Affiliations:** 1 Biodiversity Research Centre, University of British Columbia, Vancouver, Canada; 2 Universidad Nacional Agraria La Molina, Lima, Peru; 3 Universidad Nacional San Antonio Abad del Cusco, Cusco, Peru; 4 Biodiversity Research Centre, Vancouver, Canada

**Keywords:** Hummingbirds, elevational gradient, co-evolution, ornithophily, pollination ecology, Andes, Amazon, neotropics

## Abstract

**Background:**

This study establishes an altiudinal gradient, spanning from the highland Andes (2400 m) to lowland Amazon, as a productive region for the study of bird pollination in Southeastern Peru. The 'Manú Gradient' has a rich history of ornithological research, the published data and resources from which lay the groundwork for analyses of plant-bird interactions. In this preliminary expedition we documented 44 plants exhibting aspects of the bird pollination syndrome, and made field observations of hummingbird visits at three sites spanning the Manú Gradient: 2800 m (Wayqecha), 1400 m (San Pedro), and 400 m (Pantiacolla). Some of the documented plant taxa are underrepresented in the bird pollination literature and could be promising avenues for future analyses of their pollination biology. The Manú Gradient is currently the focus of a concerted, international effort to describe and study the birds in the region; we propose that this region of Southeastern Peru is a productive and perhaps underestimated system to gain insight into the ecology and evolution of bird pollination.

**New information:**

Observations were made on 11, 19, and 14 putatively bird pollinated plant species found at the high-, mid- and low-elevation sites along the gradient, respectively. Hummingbirds visited 18 of these plant species, with some plant species being visited by multiple hummingbird species or the same hummingbird species on differing occasions. Morphometric data is presented for putatively bird-pollinated plants, along with bill measurements from hummingbirds captured at each of three sites. Voucher specimens from this study are deposited in the herbaria of the Universidad Nacional de Agraria de La Molina (MOL), Peru and the University of British Columbia (UBC), Canada. The specimens collected represent a ‘snapshot’ of the diversity of bird-pollinated flora as observed over 10 day sampling windows (per site) during the breeding season for hummingbirds of Manú .

## Introduction

Manú National Park is a UNESCO Biosphere Reserve nested within the most biodiverse region in the world: the tropical Andes (Myers et al. 2000). Manú Park, and its surrounding forests encompass a remarkable elevational gradient (hereafter the 'Manú Gradient') of over 3000 m, reaching from the lowland Amazon rainforest to the Puna grasslands of the high Andes. The Manú Gradient has a rich history of ornithological research (discussed in Walker et al. 2006), and over the last decade the Manú Gradient has been the focus of numerous ornithological studies as part of the Manú Bird Project (e.g. Merkord 2010, Jankowski et al. 2012a, Jankowski et al. 2012b, Londoño et al. 2014, Londoño et al. 2016, Dehling et al. 2014, Munoz 2016). Along the gradient, tree composition and forest structure have also been described (e.g. Jankowski et al. 2012b, Malhi et al. 2010, Hillyer and Silman 2010). The wide interest in the avian community of Manú make it an ideal system for studying hummingbird pollination: population structure, range limits, and locations of uncommon and understudied hummingbirds are described and published. For example, focused studies of the high elevation Shining Sunbeam (*Aglaeactis cupripennis*) have demonstrated the effectiveness of the Manú Gradient as a study system for bird pollination (Hazlehurst et al. 2016, Hazlehurst and Karubian 2016). Therefore, the objectives of this study were to, 1) document the occurrence of putatively bird pollinated plants with voucher specimens along the Manú Gradient, 2) describe the occurrence and diversity of hummingbirds using mist-net surveys, and 3) record hummingbird visitations to flowering plants.

## Materials and Methods

**Site Selection**

We surveyed three field sites spanning an altitudinal gradient of 2400 m (400 m to 2800 m) in the southeastern Andes (Table 5): La Estación Biológica Wayqecha (Paucartambo Province, Cuzco Region, 2800 m), San Pedro (Paucartambo Province, Cuzco Region, 1400 m), and Pantiacolla (Manú Province, Madre de Dios Region, 400 m). This area is one of the most biologically rich regions in the world with an estimated species pool of nearly 1100 birds (Walker et al. 2006). To our knowledge, a comprehensive survey of the vascular plants of the region does not exist, although an increasing number of plant identification resources for this region are being made available by the Field Museum of Natural History (http://fieldguides.fieldmuseum.org). Wayqecha is characterized as high elevation cloud forest, with a mosaic of mature forest and areas with shorter trees and woody shrubs that transitions into puna grassland above treeline. San Pedro is predominately mid-montane humid rainforest, but also includes the lower extent of the montane cloud forest. Pantiacolla is situated at the interface between the Andean foothills and the lowland Amazon. Detailed environmental characteristics for these sites have been summarized in Malhi et al. (2016). Sampling was carried out between September 4, 2016 and October 13, 2016, falling within the avian montane breeding season. A distinct rainy season occurs from November through April and a dry season from May through August. Annual precipitation for higher elevations (2700-3000 m) ranges from 1700-2000 mm (Girardin et al. 2010) and is generally >2000 mm for lowland (100-400 m elevation) sites (Rapp and Silman 2012). Time constraints afforded less than two weeks (10.3 ± 2.1 days) for botanical and avian sampling at each site.

**Data Collection**

Pre-cut singletrack trails were used to access sampling areas away from the Manú Road (main access road that runs along the southeastern border of Manú National Park). We sampled hummingbirds using standard (12 x 3 m, 34 mm mesh) mist-nets along trail systems only. Mist-netting sites were sampled during the primary breeding season (August–November) for two consecutive days from approximately 0600–1200 hrs during suitable weather conditions (i.e., no periods of extended heavy rain, high winds, or other situations that could compromise researcher or bird safety). Each site consisted of an array of ten to fifteen nets placed in forested and open habitat and spaced at intervals of 25-50 m. Ten sites were sampled at Wayqecha and San Pedro, and 8 sites were sampled at Pantiacolla. Hummingbird bill length was measured from the bill tip to the nares. Bill width was measured from the anterior edge of the nares. All captured hummingbirds were marked by cutting the terminal 1-2 cm of one rectrix to avoid resampling of individuals.

Both trails and the Manú Road were used to opportunistically collect plants. Plants were considered putatively bird pollinated if they met criteria adhering to typical bird pollination ‘syndromes’; namely, dilute nectar and long tubular flowers (Fenster et al. 2004), though we acknowledge the limitations of surveying by these critera (Ollerton et al. 2009). Plants of interest were photographed, their location marked using a hand-held Garmin 64s global positioning system, and a description of the immediate habitat recorded. We then measured nectar concentration of mature flowers (Sper Scientific no. 66214-988), recorded corolla dimensions and colour (by visual inspection), and processed each plant using standard herbarium techniques Bridson and Forman 2000) (SERFOR collection permit no. 343-2016). All dried and pressed specimens are deposited at the herbaria of the Universidad Nacional Agraria La Molina (MOL), Peru and the University of British Columbia (UBC), Canada (SERFOR export permit no. 09125-2017).

## Results

We identified 44 putatively bird pollinated plants of interest belonging to 16 families (Table 1, Figs 1, 2, 3, 4, 5). Corolla length and width of sampled plants ranged from 8-120 mm (x̅ = 39.7 ± 27.4, n = 42) and 1-60 mm (x̅ = 11.4 ± 12.2), respectively. We measured nectar concentration for 11 of these species. In each case, nectar concentrations fell within a typical bird pollination syndrome (Stiles 1978, Fenster et al. 2004), ranging from 12-25.5% (Table 1). Corolla colour and immediate habitat characteristics were recorded for each plant (Table 1, see also Table 5).

We recorded 23 hummingbird visitations to 18 plant taxa belonging to 12 plant families (Table 2). Bill length and width of sampled hummingbirds ranged from 11.5-39.6 mm (x̅ = 24.3 ± 7.6, n = 41) and 2.5-3.0 mm (x̅ = 2.9 ± 0.4, n = 40), respectively (Figs 6, 7, 8, Table 3).

Diversity of plants exhibiting the bird pollination syndrome does not differ across the gradient in the time frame sampled (Table 4).

## Discussion

Hummingbird pollination is common and well-established in Neotropical montane and lowland environments. Our observations and collected specimens exemplify that bird-plant interactions are readily observed along the Manú Gradient - an area that is relatively accessible has been subject to only a handful of studies on hummingbird pollination (*Oreocallis grandiflora*, Proteaceae; Hazlehurst et al. 2016, Hazlehurst and Karubian 2016).

Along the gradient, putatively bird pollinated plants were generally characterized by long corollas and were predominantly coloured red, yellow, orange, or some combination thereof. Previous documentation of bird pollination exists for each of the 16 families collected (Cronk and Ojeda 2008, Johnson and Nicolson 2008), but undocumented species-level bird pollination systems may arise from focusing on lesser-stuided taxa (e.g. *Thyrsacanthus*, *Pentagonia*, *Pachystachys*). Many putatively bird pollinated plants contained too little nectar to effectively measure sugar concentration at the time of sampling. We suspect that early morning visitations by nectarivorous birds and insects (i.e., both pollinators and nectar robbers) influenced this outcome. Indeed, in some cases inspection of certain plants revealed that the flower had been recently robbed as indicated by punctures at the base of the corolla. In as little as bird pollination has been studied along the Manú Gradient, even less is known of the ecological and evolutionary dynamics of nectary robbery. As this survey was preliminary, time did not allow for multi-day sampling at one locale to isolate nectar. A focus on a specific plant taxon would allow familiarity for nectar phenology and hence, more effective collection of nectar.

We recorded 23 independent visits by hummingbirds to 19 different plant taxa over 33 days. These observations by no means represent a comprehensive list of the total diversity for hummingbirds (Walker et al. 2006), bird-pollinated plants, or the interactions between these two groups. An estimate of total diversity will come only with an extended sampling effort at each site. Relatively few hummingbirds were captured or observed in the lowlands (Table 3) compared to the other two sites. It is likely that this resulted from differences in foraging behavior between hummingbird species, rather than local abundance. For example, in the lowlands, a higher proportion of hummingbirds (e.g. *Phaethornis*) exhibit traplining behaviour (i.e. repeated visits along a route of flowering locations) compared to territorial guarding of floral resources. In addition, because of the higher canopy, many of the trees, lianas, and epiphytes inhabit canopy heights that are logistically difficult to sample.

The number of plants exhibiting bird pollination syndrome and number of bird visits observed are comparable between sites. That is, at a coarse scale we did not find any indication that elevation affects the absolute diversity of bird pollinated plant taxa (as expected by Cruden 1972), although the Manú Gradient would be an ideal location to test the hypothesis that bird and insect pollinated plants occupy distinct ecological niches. Between species, corolla length and width encompasses a great amount of variation, but hummingbird bill morphology varies less Tables 1, 3. This may speak to the adaptability of flowers relative to bills. It may be that because flowers serve a singular purpose (attraction and exclusion of pollinators and robbers, respectively), whereas bills have many uses (feeding, aggression, preening, balance), that bill evolution is relatively constrained. Bill morphology data will be used to inform phylogenetic tests of bill-flower shape evolution in future studies.

Evaluating the extent to which plants and their pollinators contribute to maintaining local biodiversity, and identifying keystone species within these systems (Ebenman and Jonsson 2005) will be important to maintaining ecological and cultural heritage in the Manú region (Ministerio del Ambiente (Ministry of Environment) 2017). This study provides a baseline for future work in pollination ecology along the Manú Gradient. Any one of the 44 plant species highlighted here warrants closer investigation, and we anticipate that further studies will help clarify the roles of hummingbirds as pollinators for the plant taxa described herein..

## Figures and Tables

**Figure 1a.**
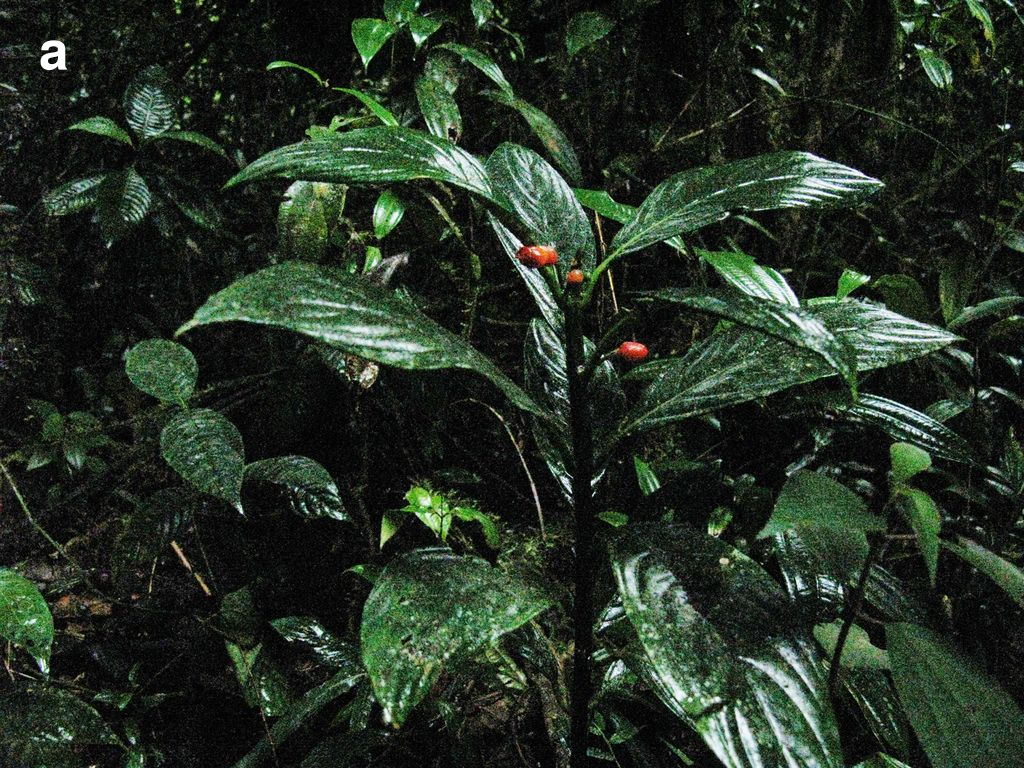
MMAB 1 (*Besleria* sp. 1)

**Figure 1b.**
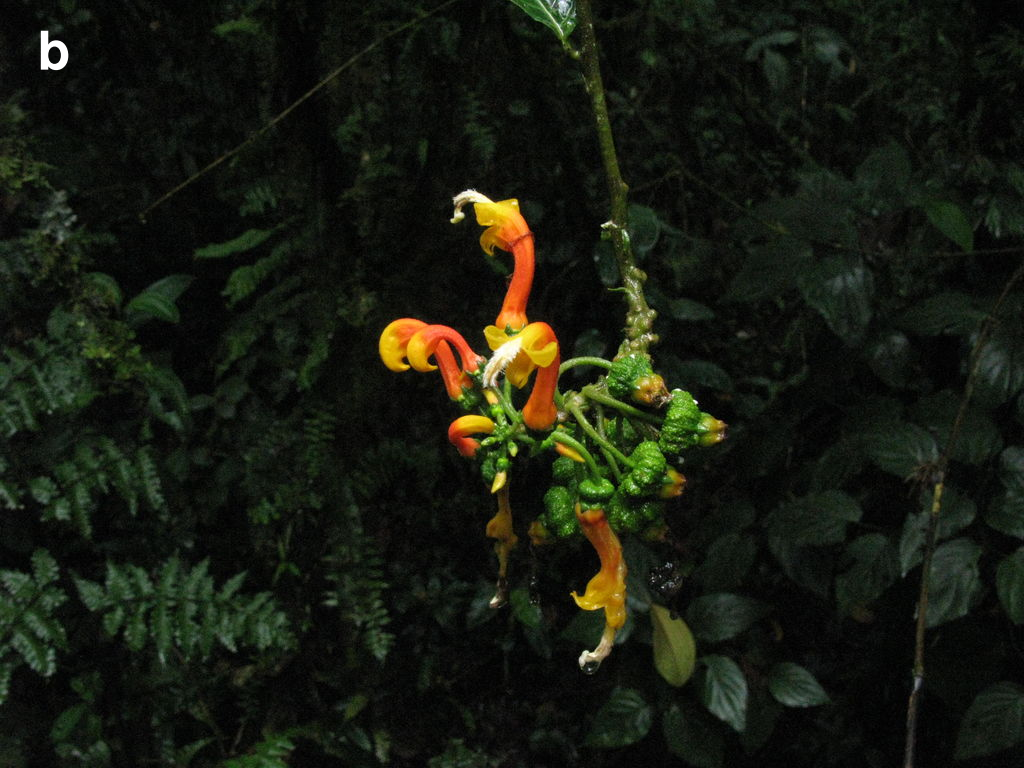
MMAB 2, 3 (*Centropogon granulosus*)

**Figure 1c.**
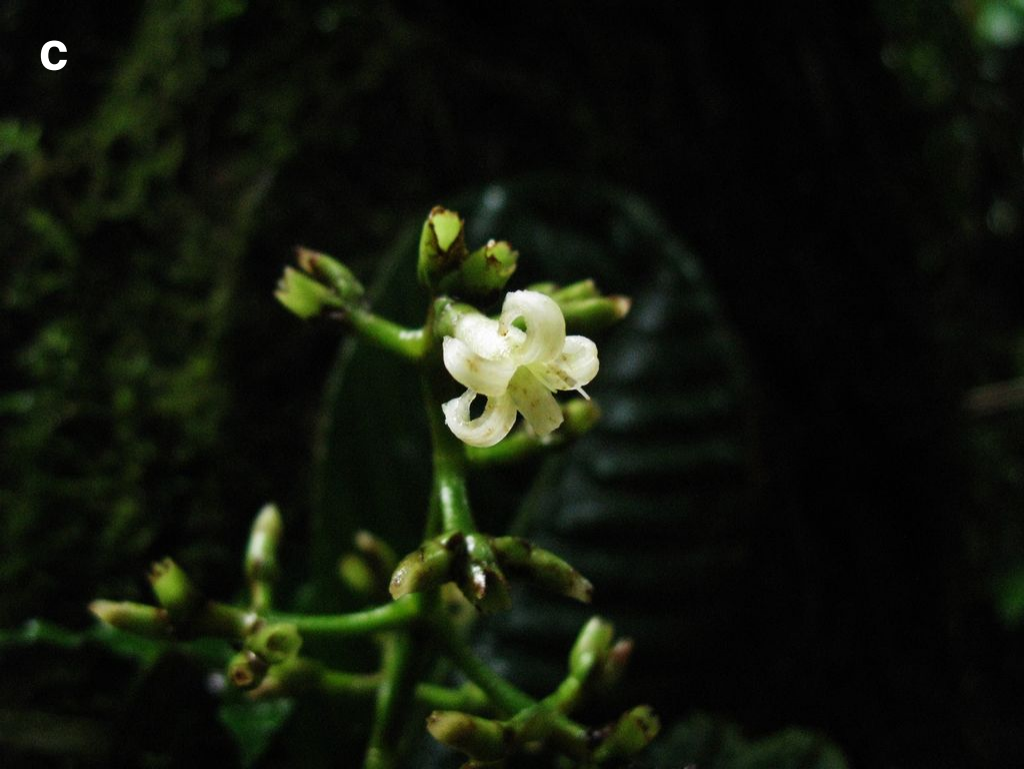
MMAB 6, 7 (*Miconia* sp. 1)

**Figure 1d.**
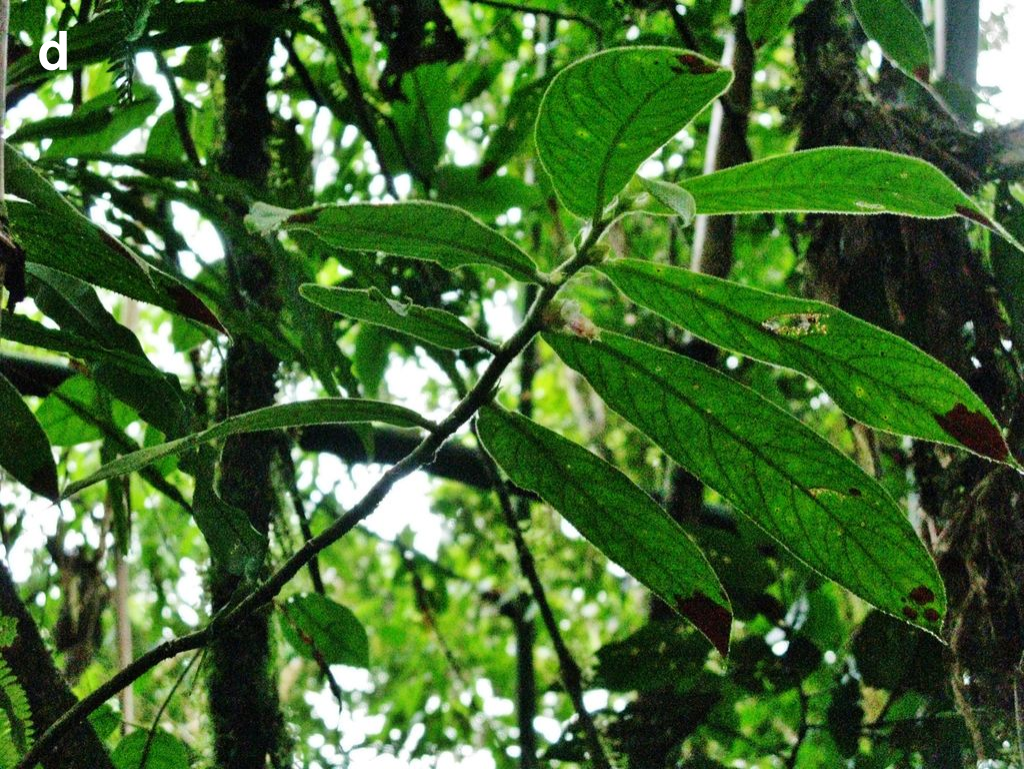
MMAB 8, 9 (Columnea aff. shimpfii)

**Figure 1e.**
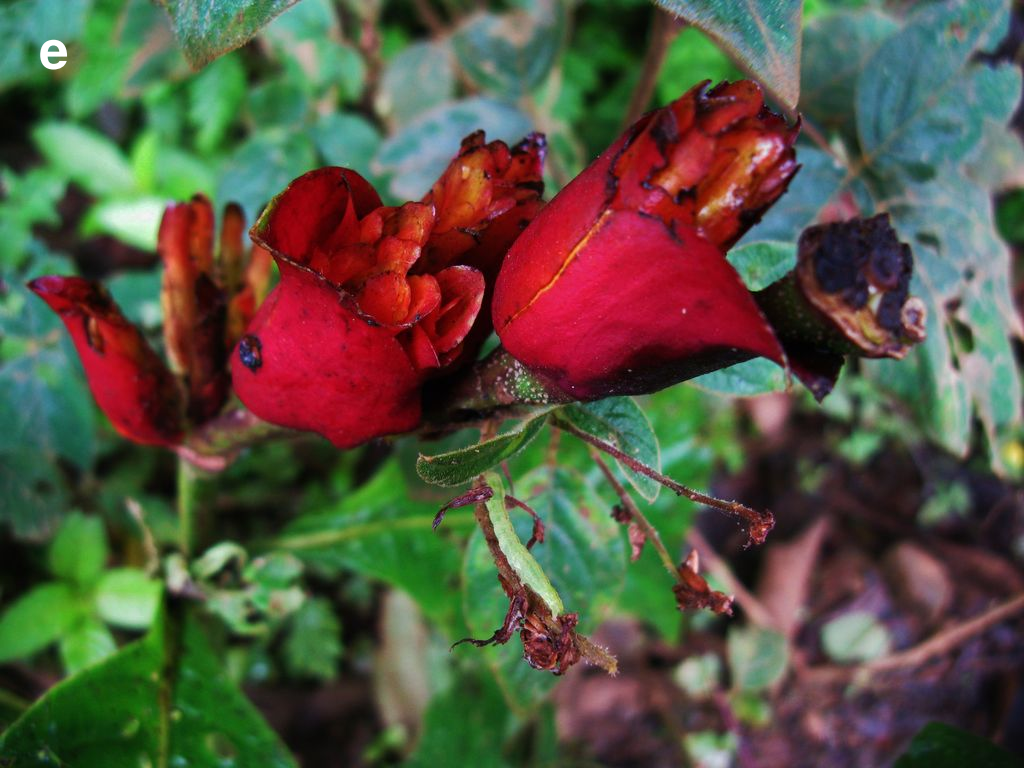
MMAB 20, 21 (*Sanchezia* sp. 1)

**Figure 1f.**
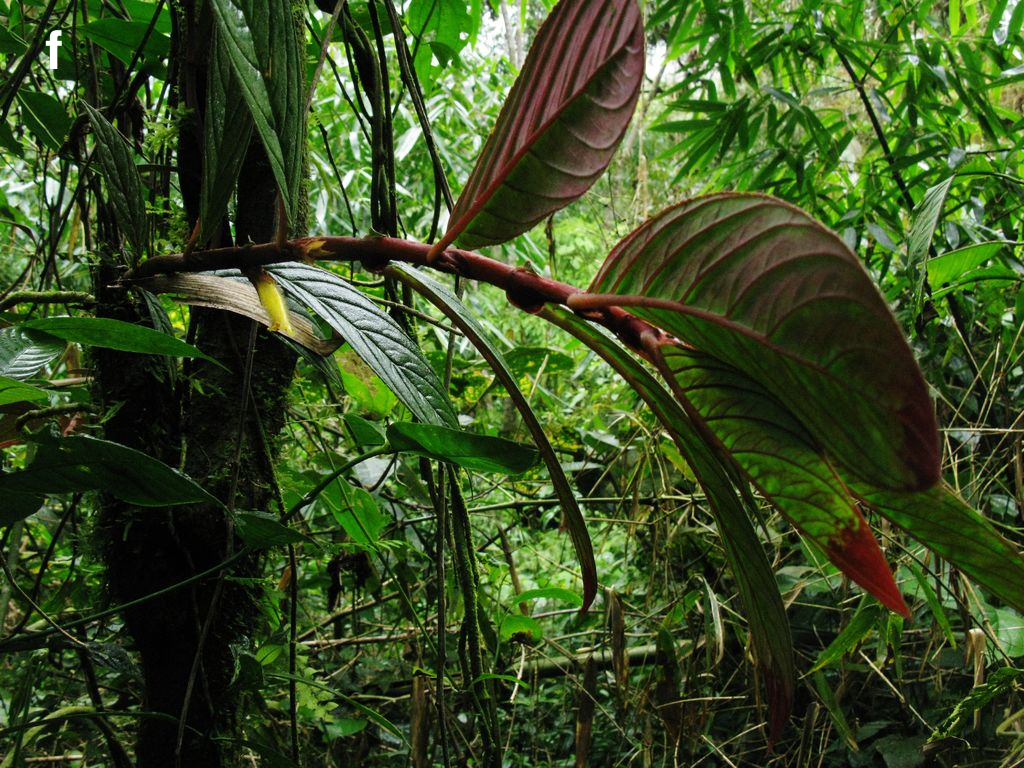
MMAB 22, 23 (*Columnea guttata*)

**Figure 2a.**
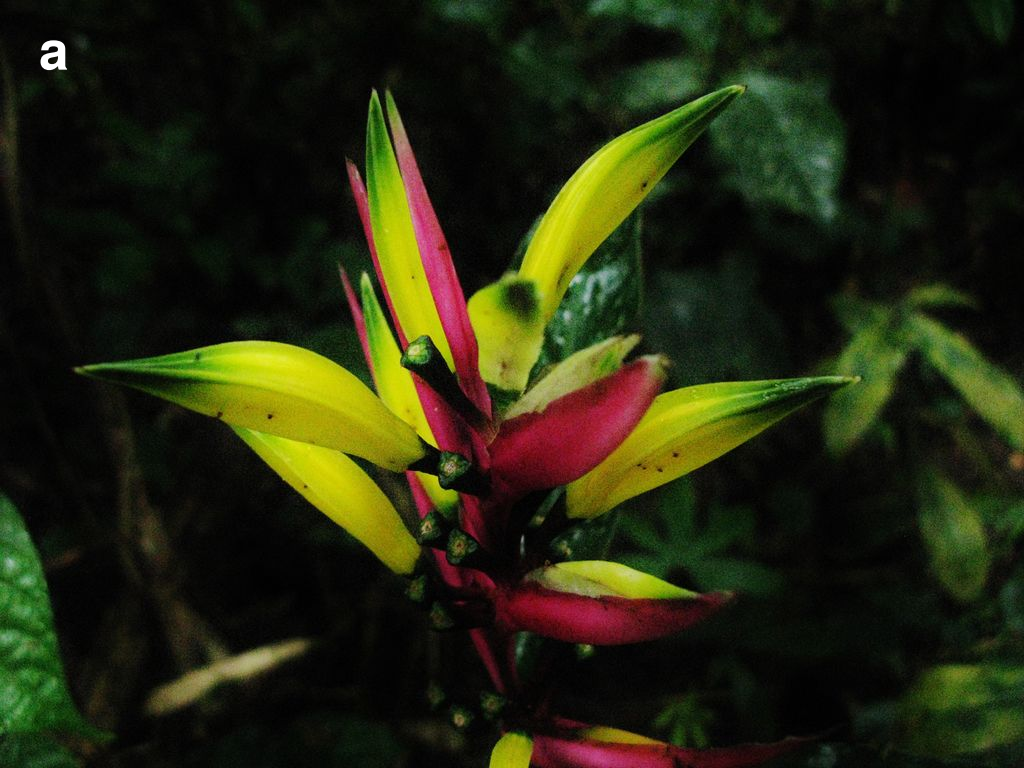
MMAB 24, 25 (*Heliconia subulata*)

**Figure 2b.**
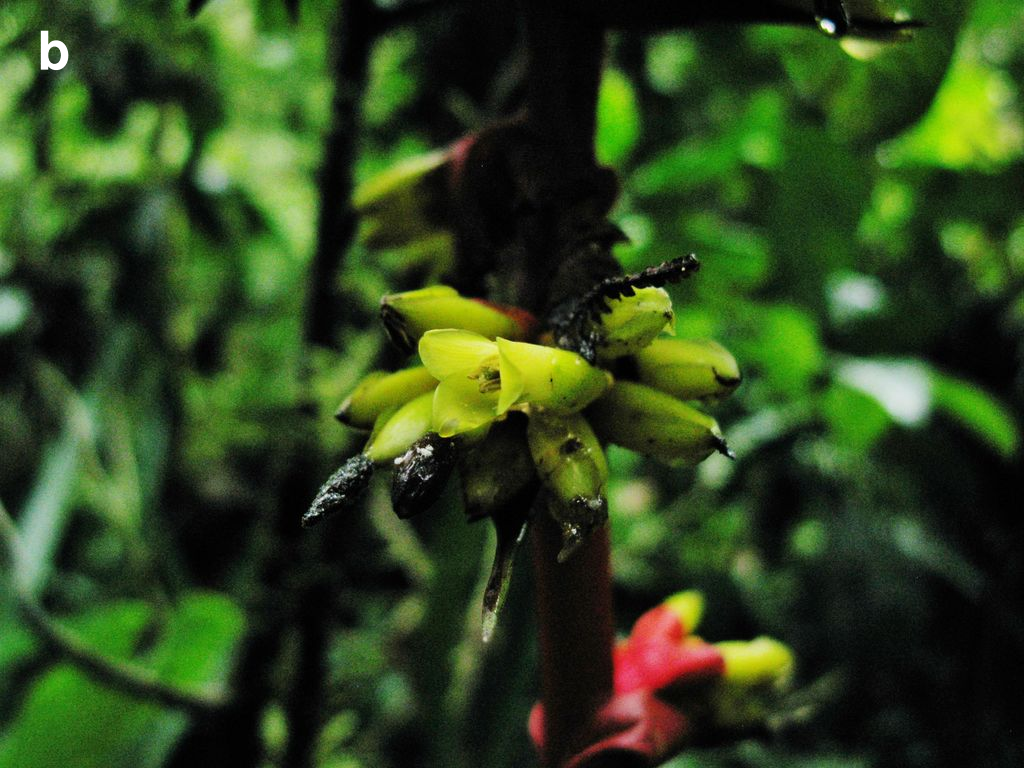
MMAB 26, 27 (*Guzmania weberbaueri*)

**Figure 2c.**
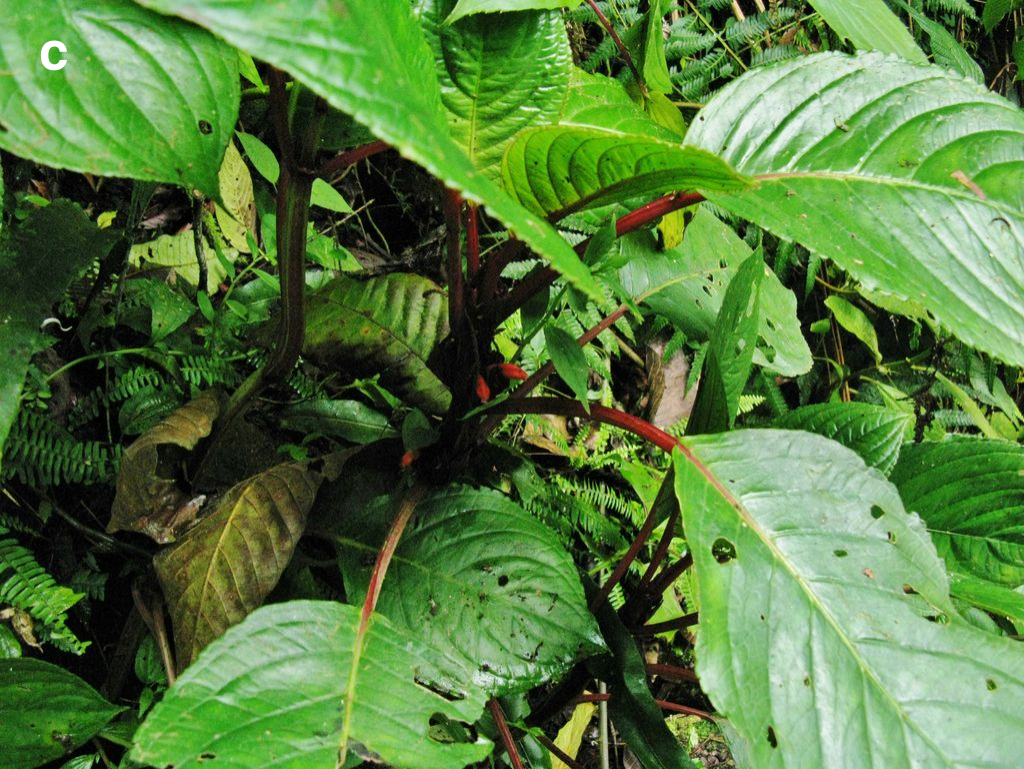
MMAB 39, 40 (*Drymonia urceolata*)

**Figure 2d.**
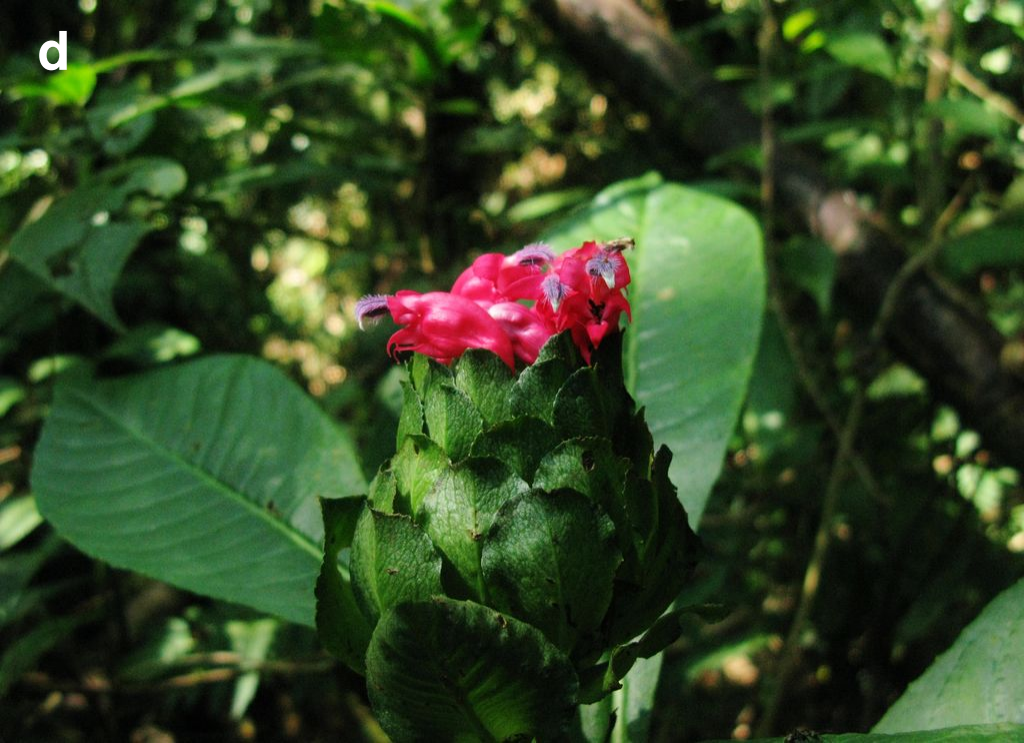
MMAB 45 (*Centropogon congestus*)

**Figure 2e.**
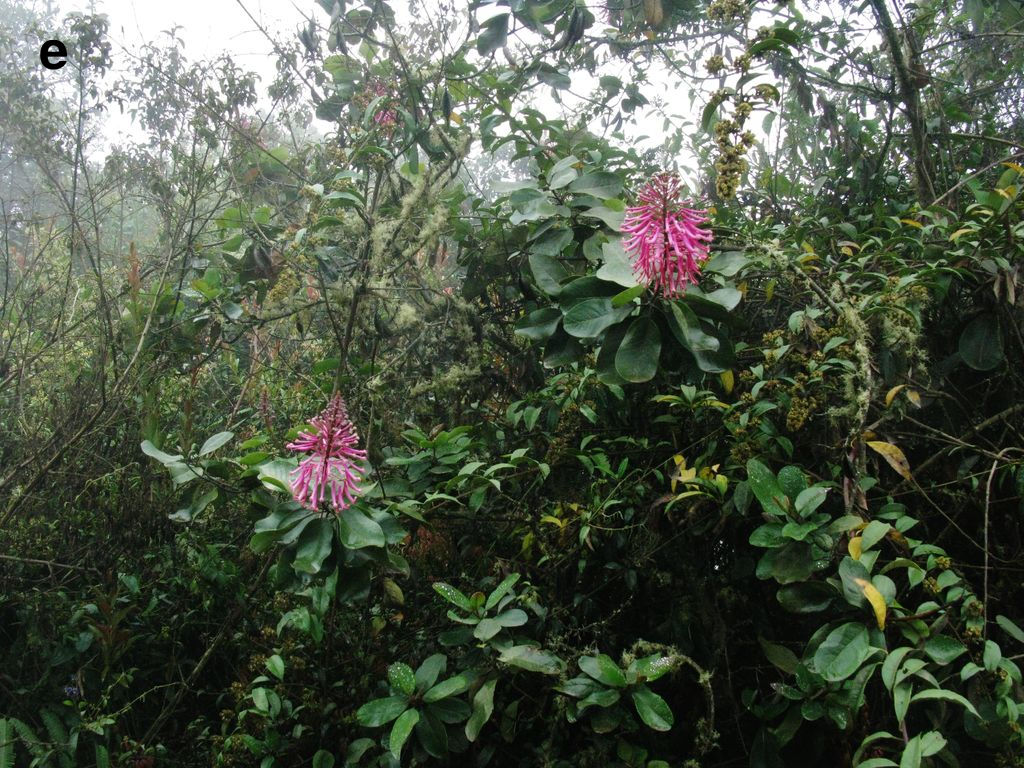
MMAB 48, 49 (*Oreocallis grandiflora*)

**Figure 2f.**
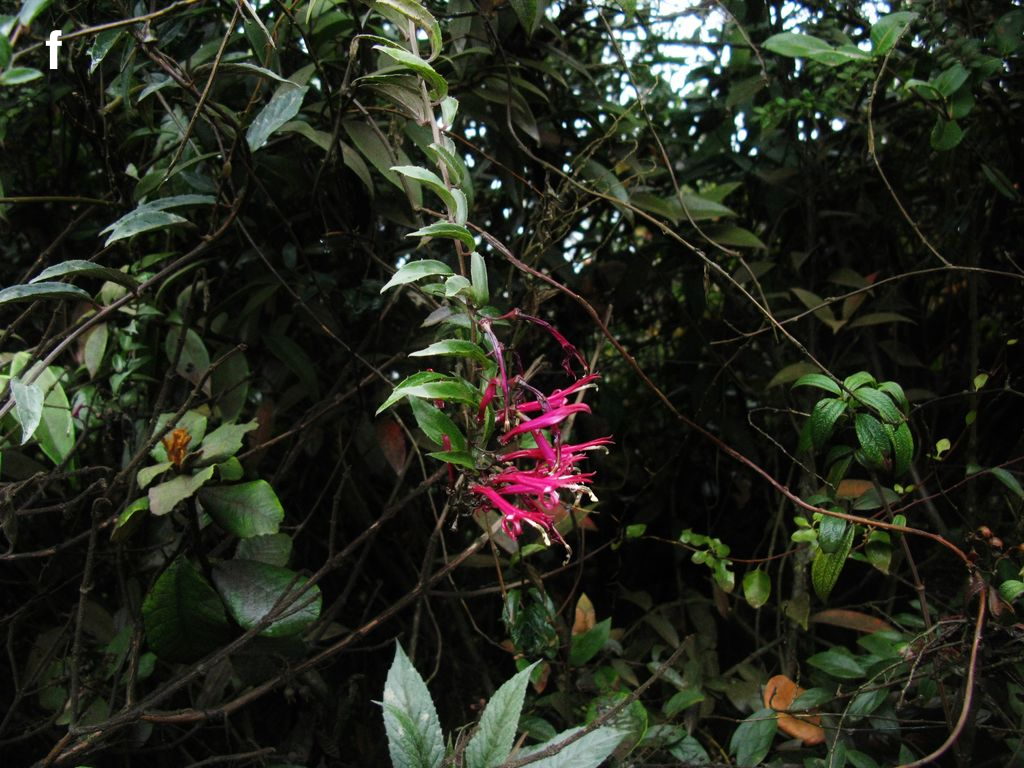
MMAB 50, 51 (*Siphocampylus scandens*)

**Figure 3a.**
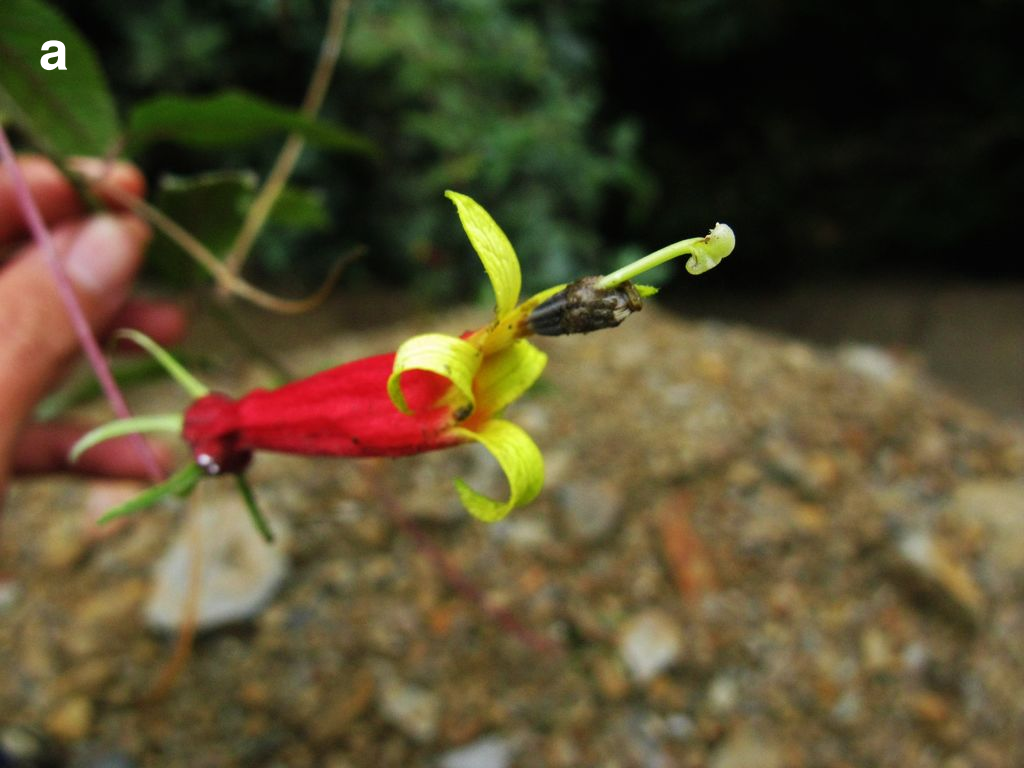
MMAB 52, 53 (*Siphocampylus orbignianus*)

**Figure 3b.**
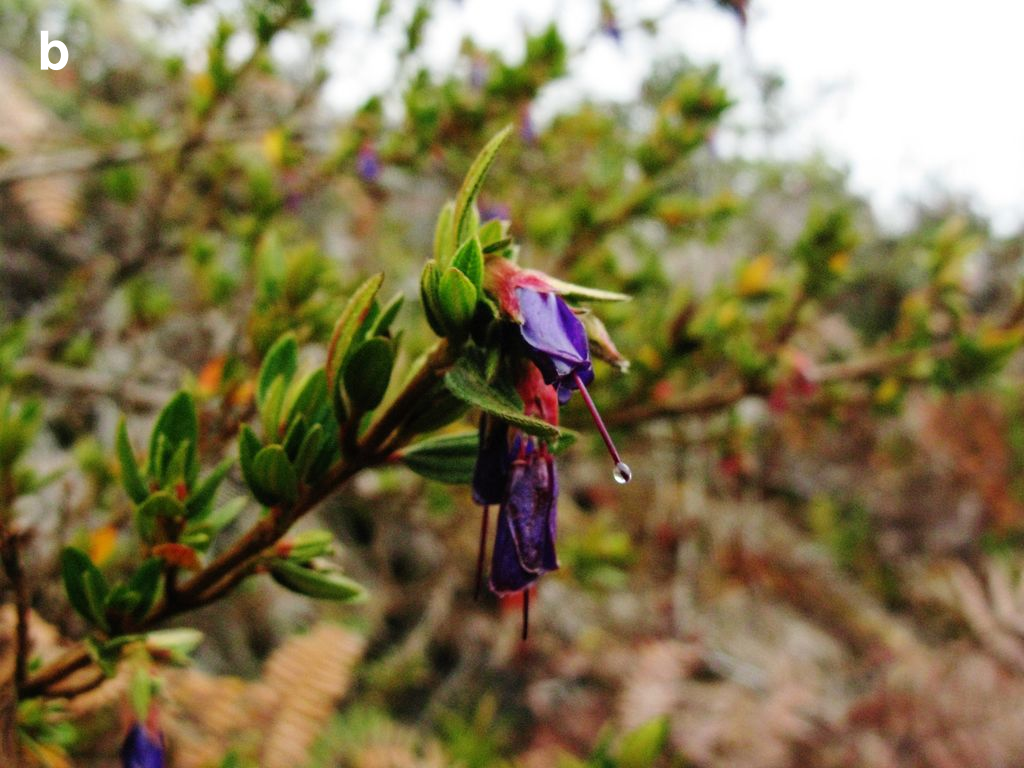
MMAB 54, 55 (*Brachyotum rostratum*)

**Figure 3c.**
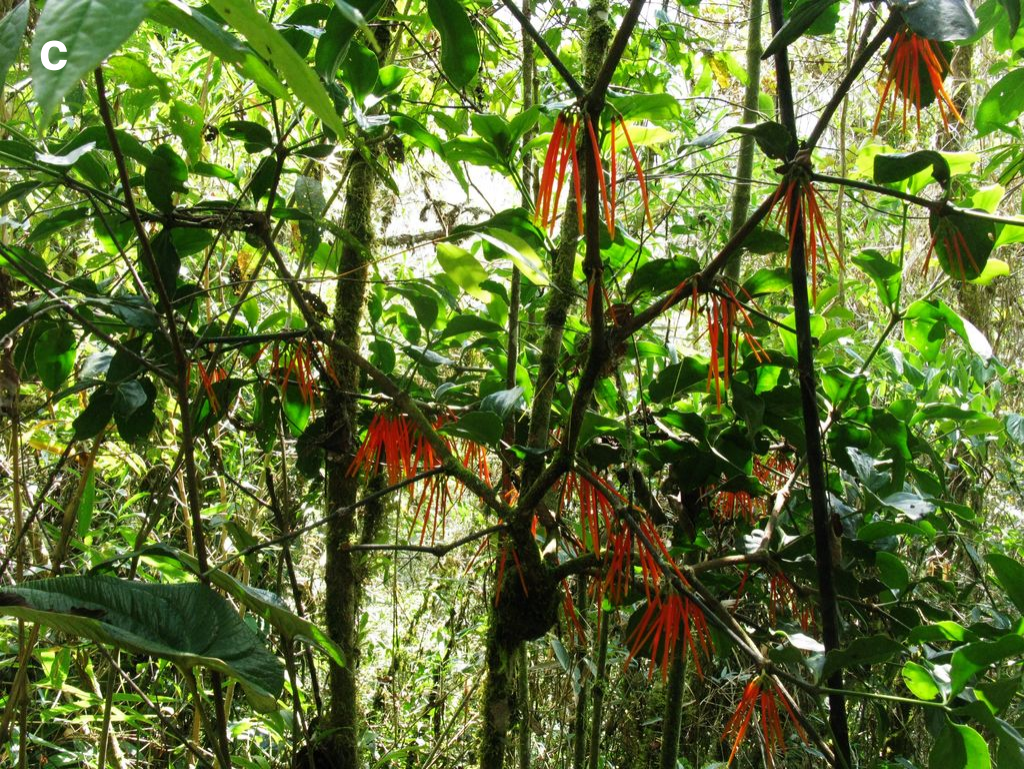
MMAB 56, 57 (*Aetanthus nodosus*)

**Figure 3d.**
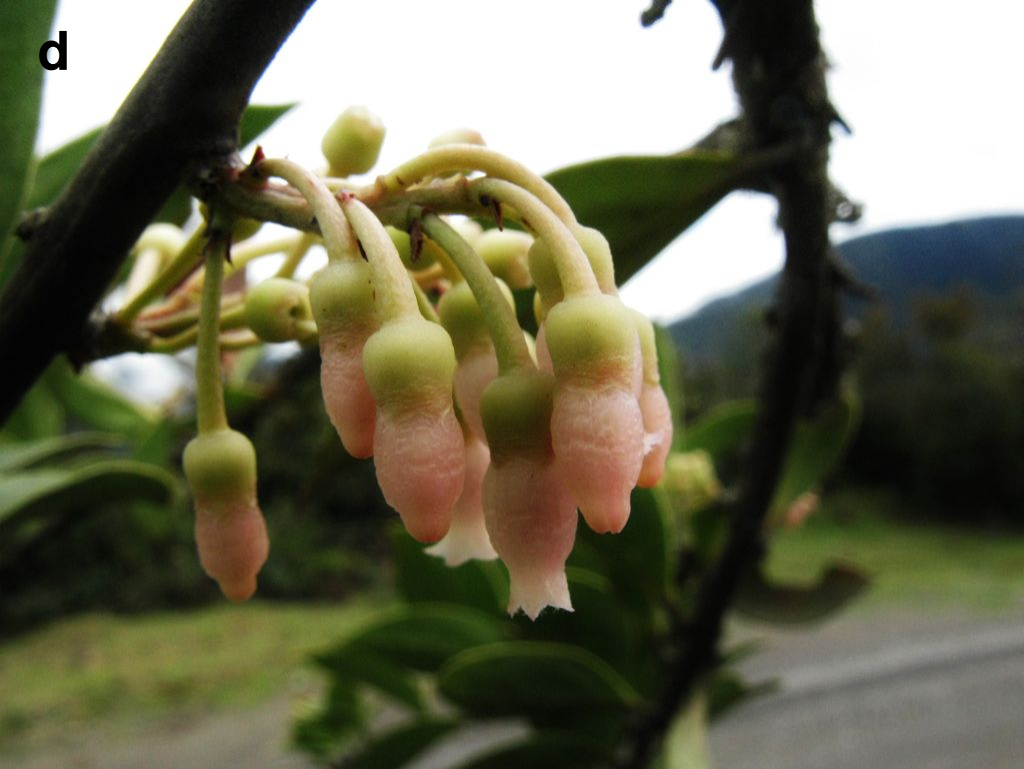
MMAB 58, 59 (*Gaultheria* sp. 1)

**Figure 3e.**
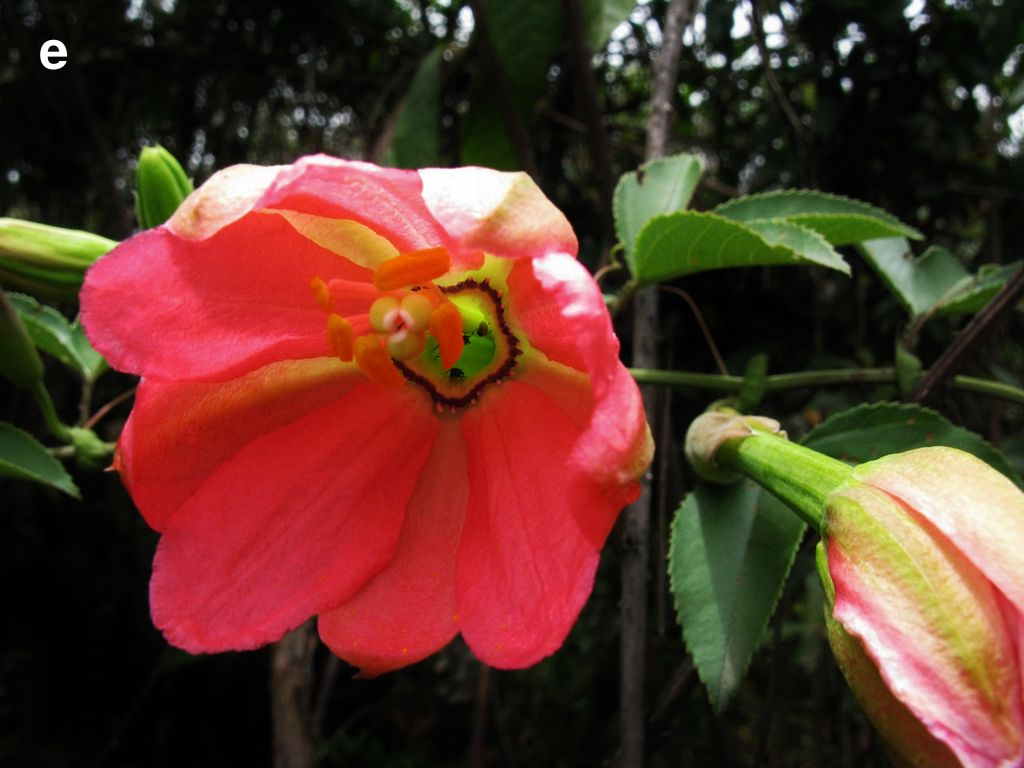
MMAB 60, 61 (*Passiflora mixta*)

**Figure 3f.**
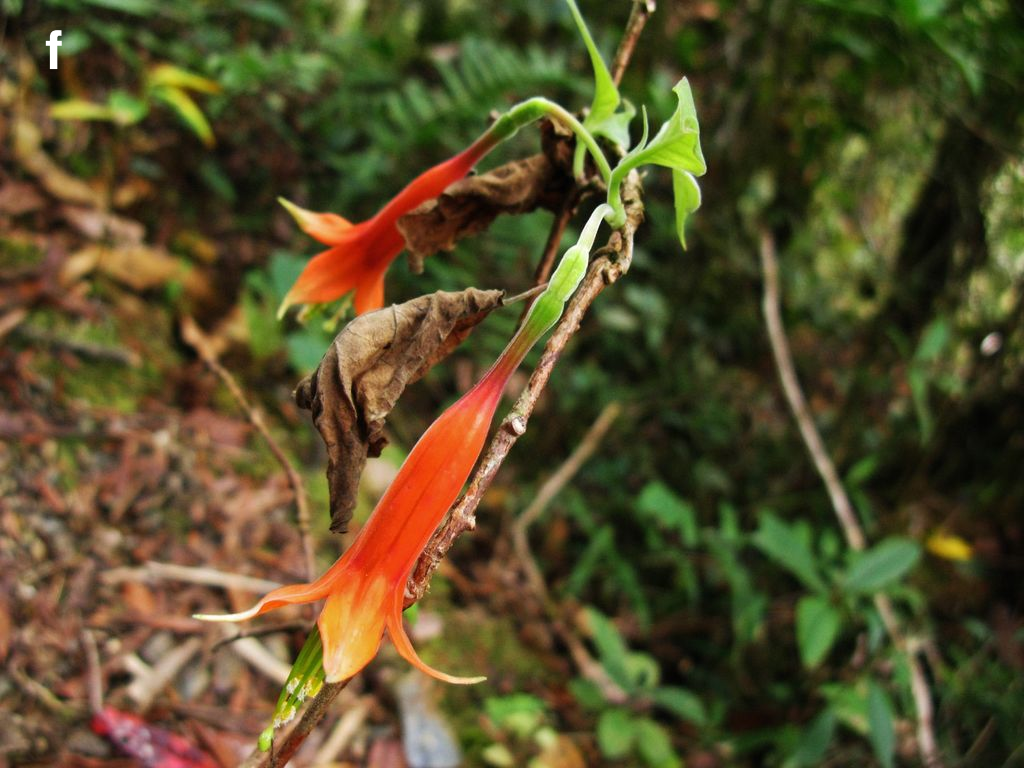
MMAB 62, 63 (*Fuchsia* sp. 1)

**Figure 4a.**
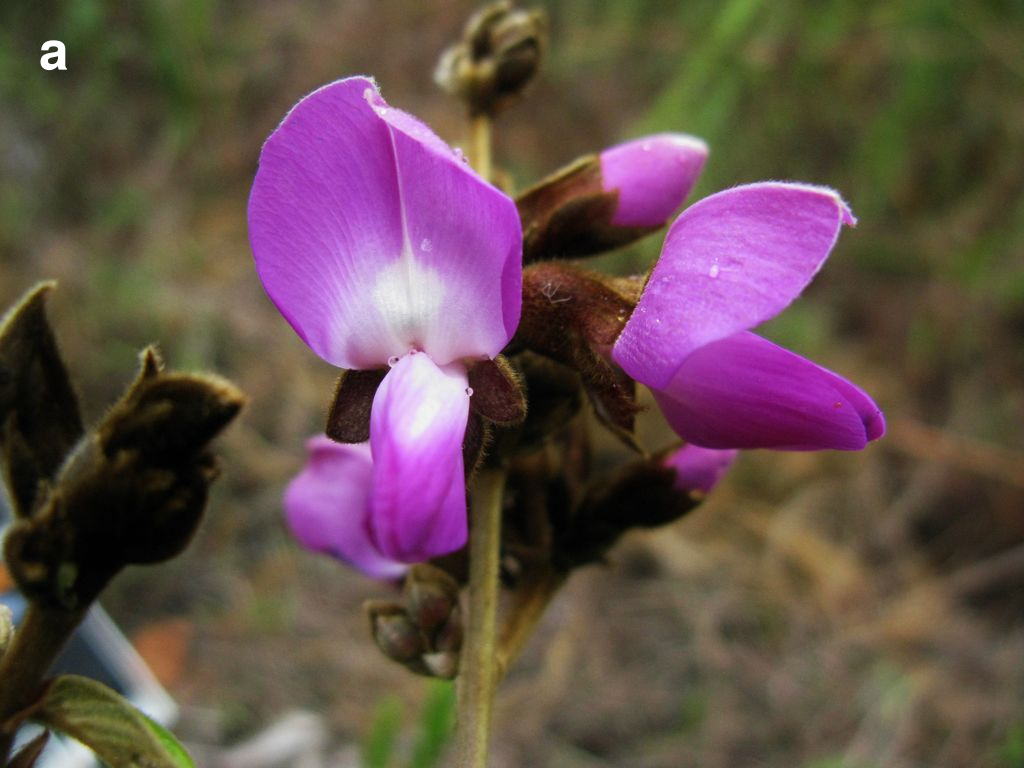
MMAB 64, 65 (*Desmodium* sp. 1)

**Figure 4b.**
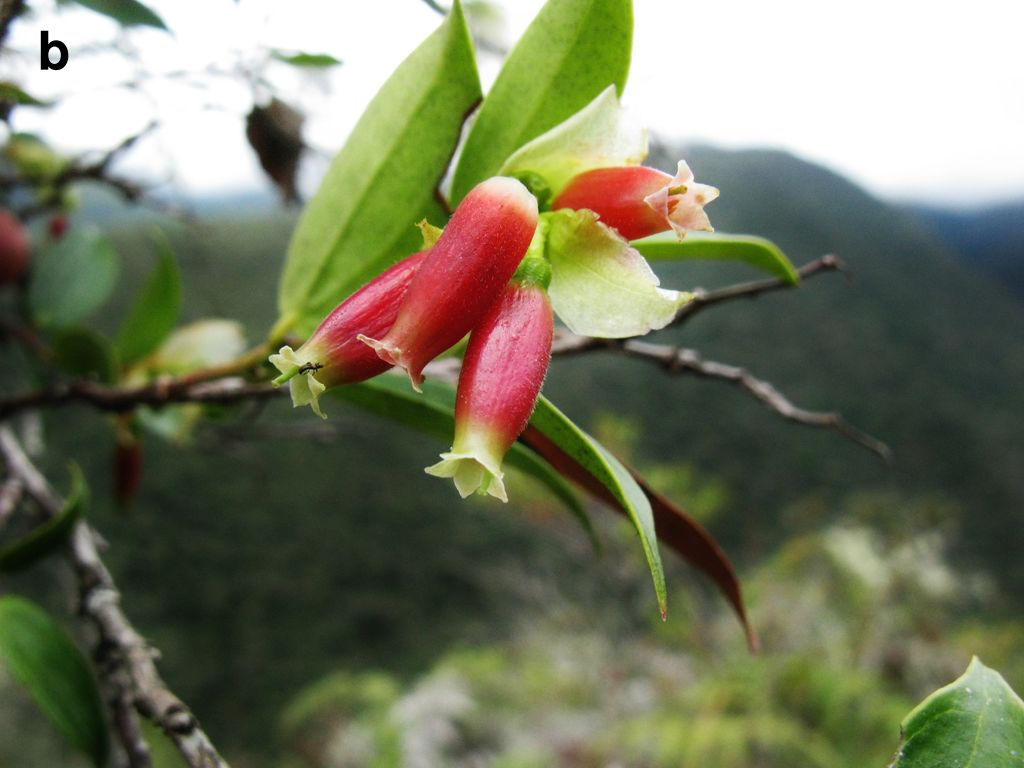
MMAB 66, 67 (*Siphonandra* sp. 2)

**Figure 4c.**
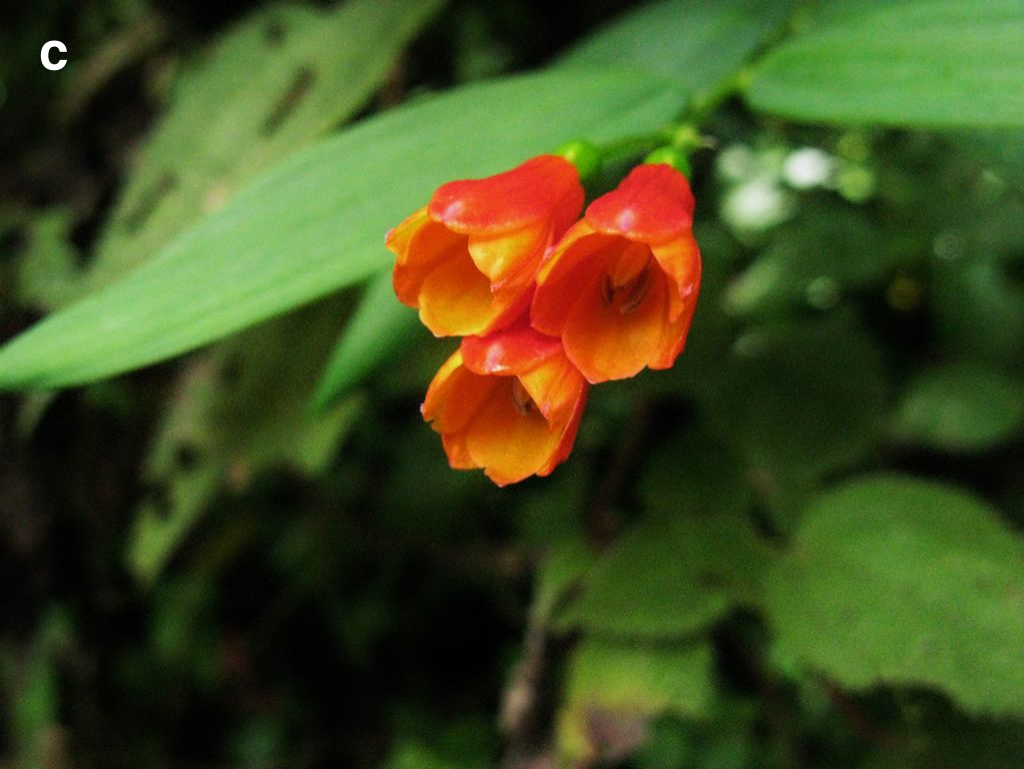
MMAB 68, 69 (*Bomarea* sp. 1)

**Figure 4d.**
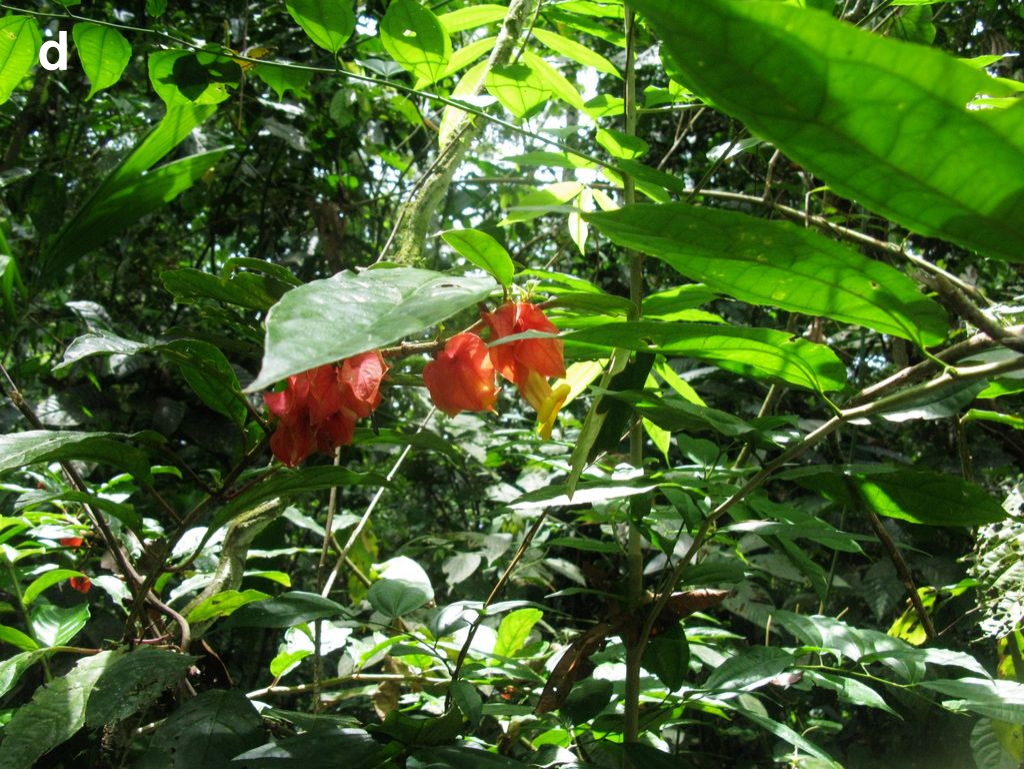
MMAB 70, 71 (*Drymonia semicordata*)

**Figure 4e.**
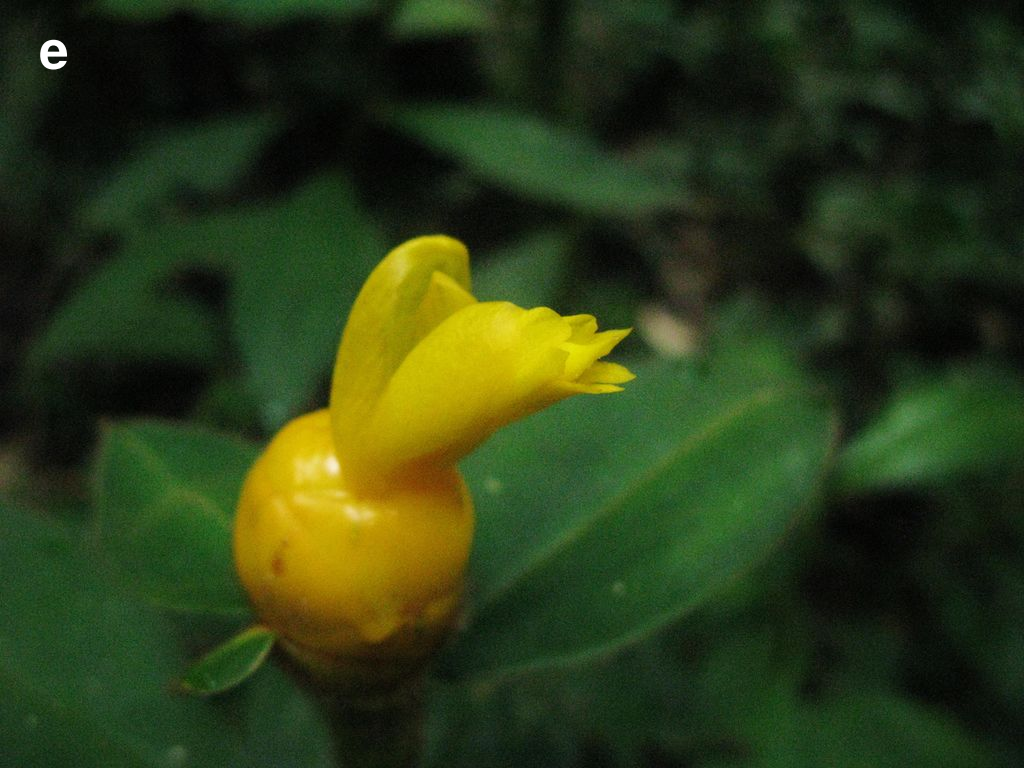
MMAB 74, 75 (*Costus* sp. 3)

**Figure 4f.**
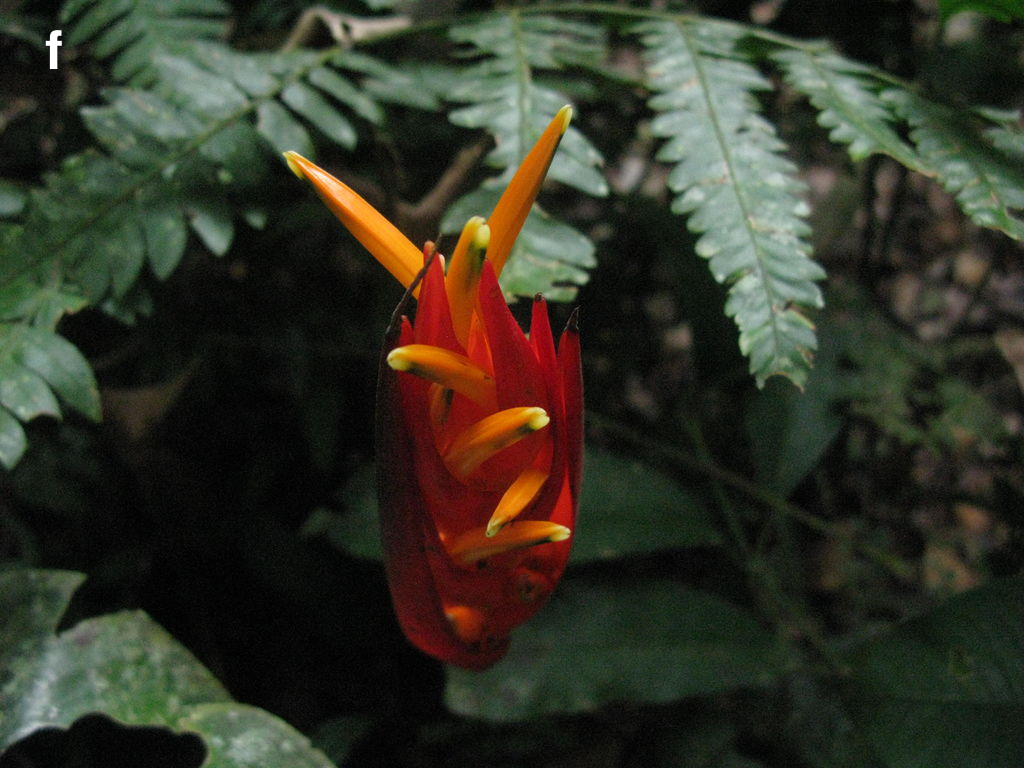
MMAB 76, 77 (*Heliconia densiflora*)

**Figure 5a.**
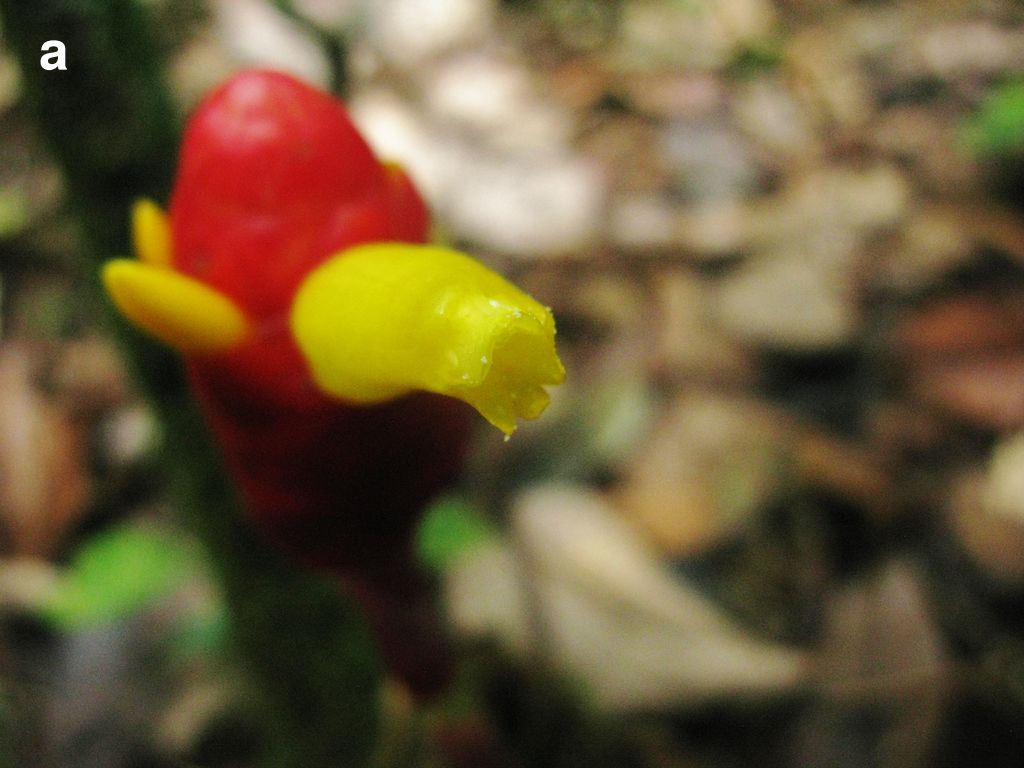
MMAB 78, 79 (*Costus* sp. 2)

**Figure 5b.**
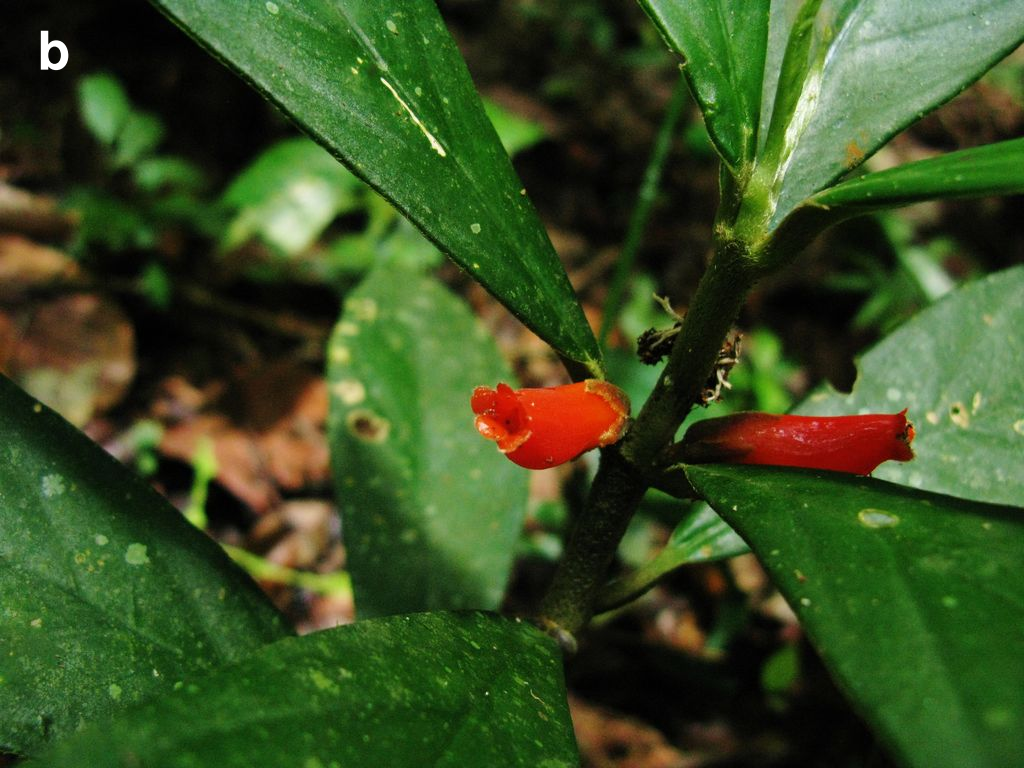
MMAB 80, 81 (*Besleria* sp.4)

**Figure 5c.**
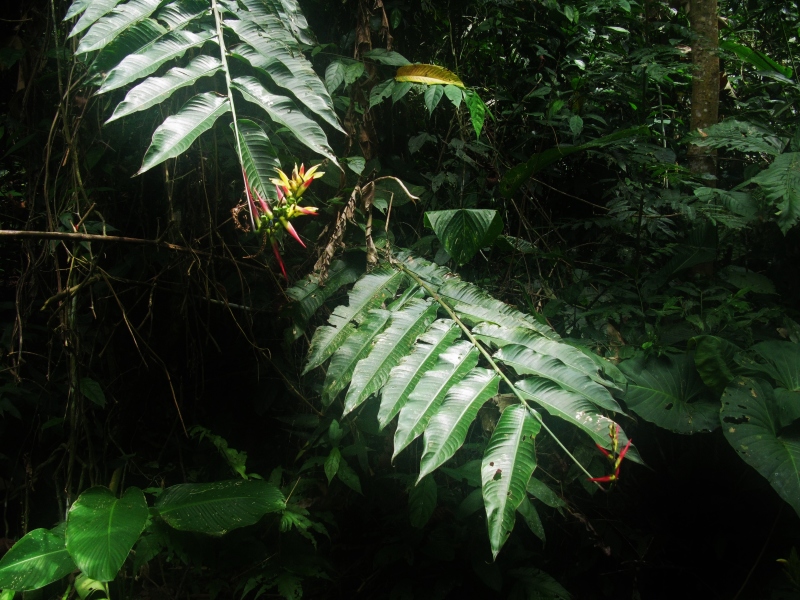
MMAB 82, 83 (*Heliconia schumanniana*)

**Figure 5d.**
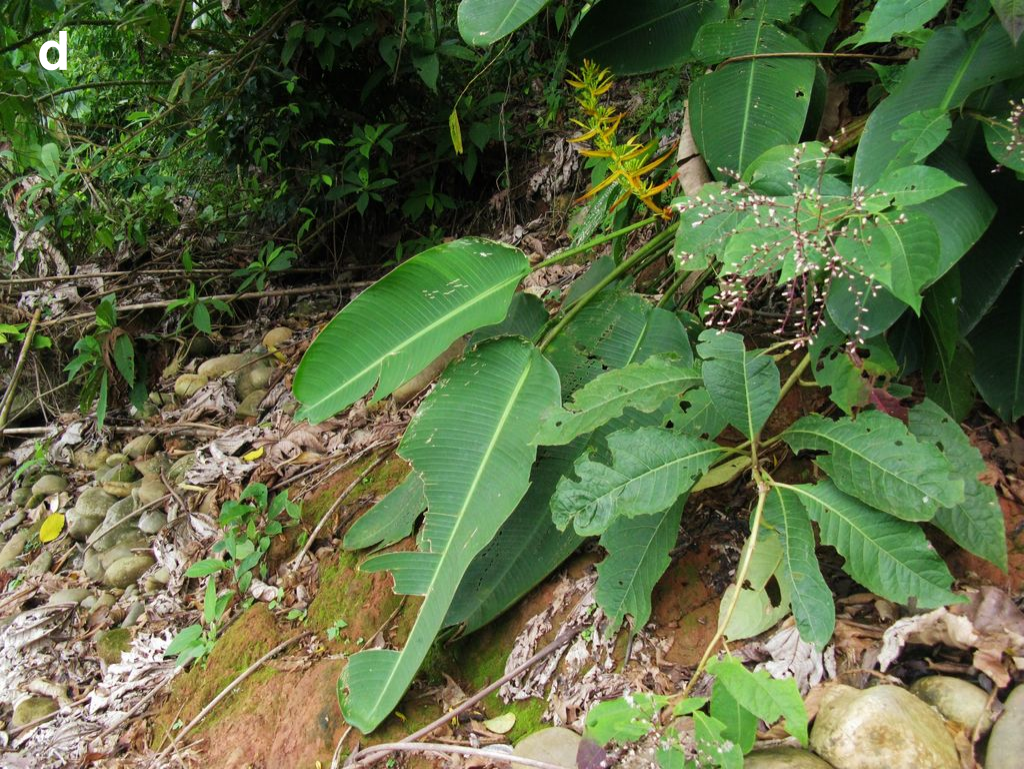
MMAB 84, 85 (*Heliconia lingulata*)

**Figure 5e.**
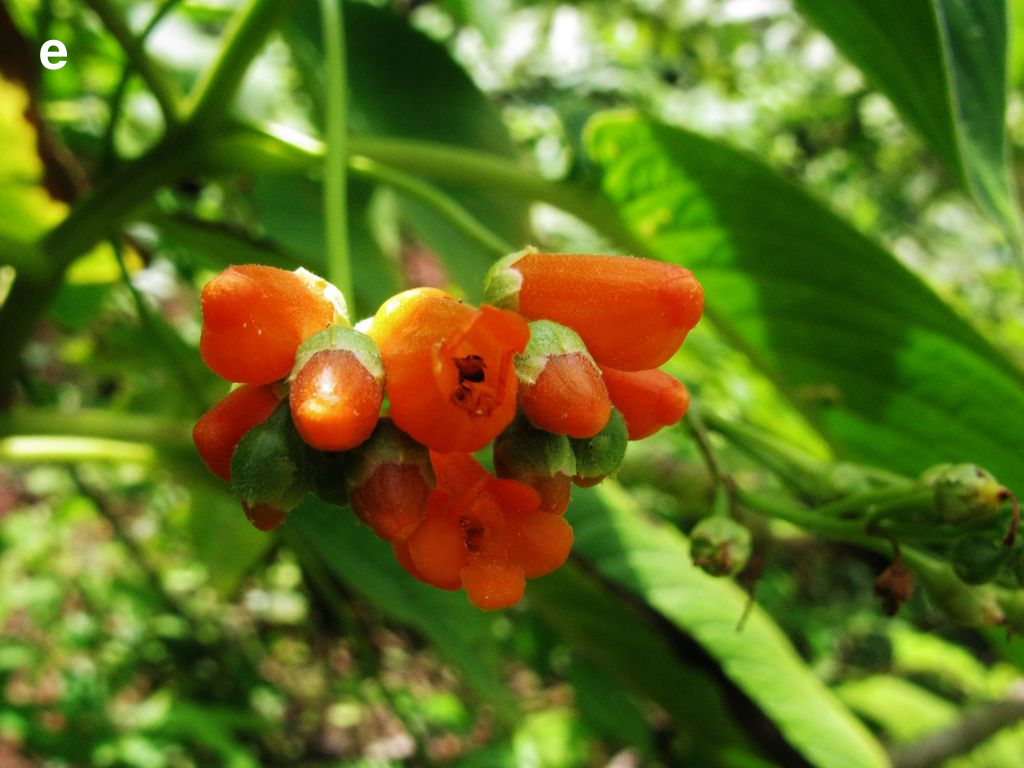
MMAB 86, 87 (*Besleria* sp. 2)

**Figure 5f.**
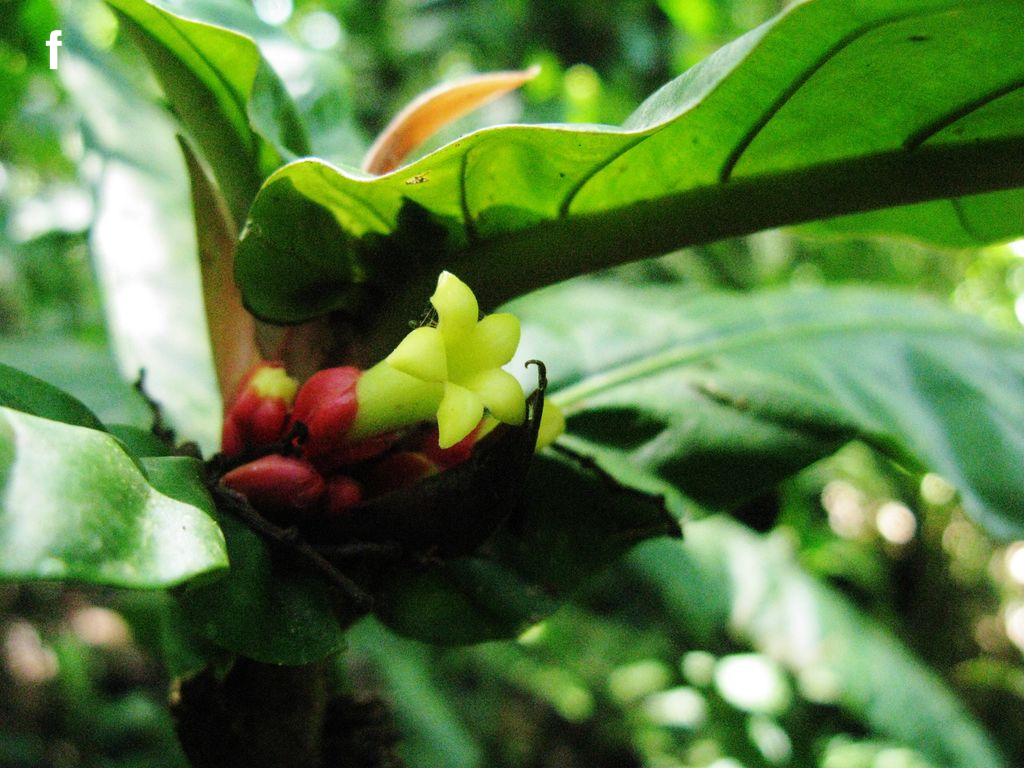
MMAB 92, 93 (*Pentagonia* sp. 1)

**Figure 6a.**
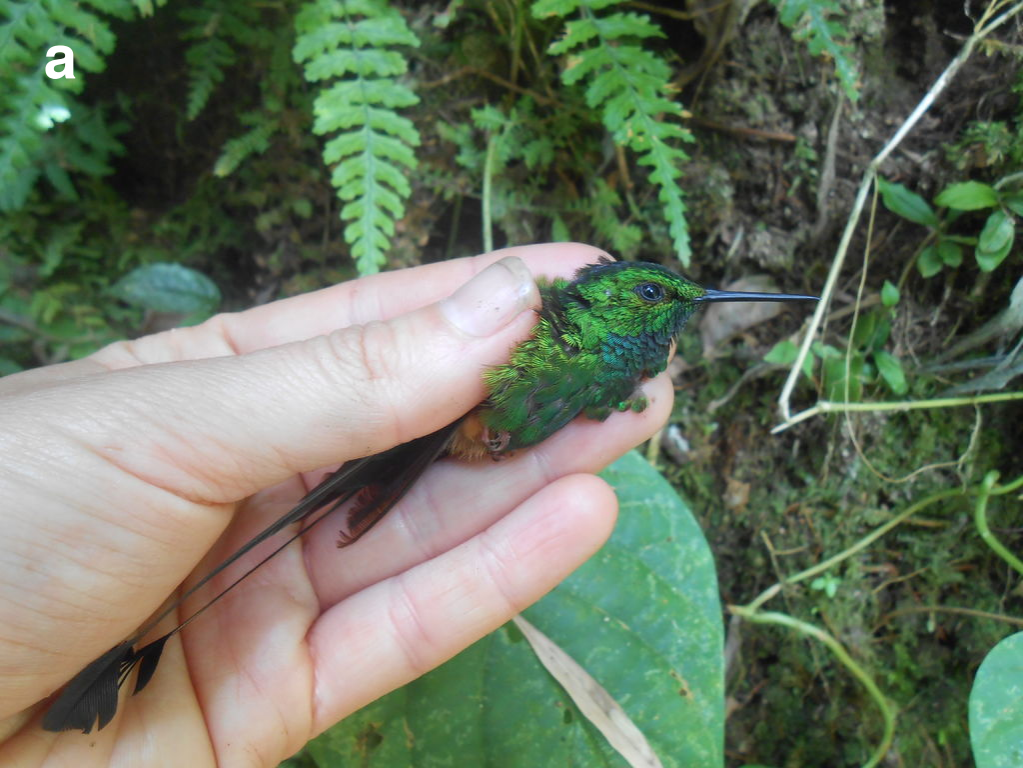
Booted Racket-tail (*Ocreatus underwoodii*)

**Figure 6b.**
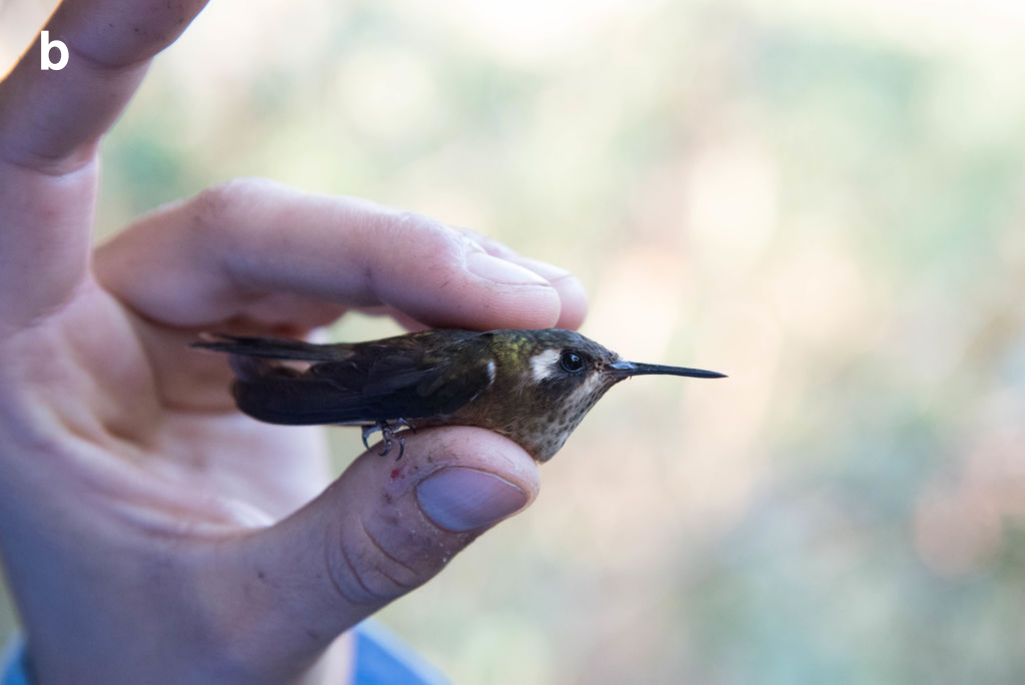
Speckled Hummingbird (*Adelomyia melanogenys*)

**Figure 7a.**
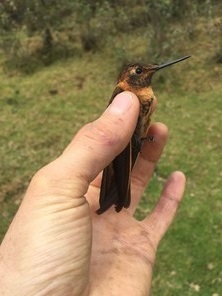
Shining Sunbeam (*Aglaeactis cupripennis*)

**Figure 7b.**
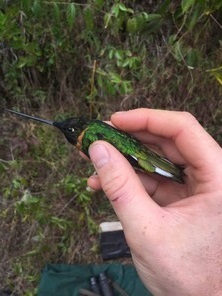
Collared Inca (*Coeligena torquata*)

**Figure 8a.**
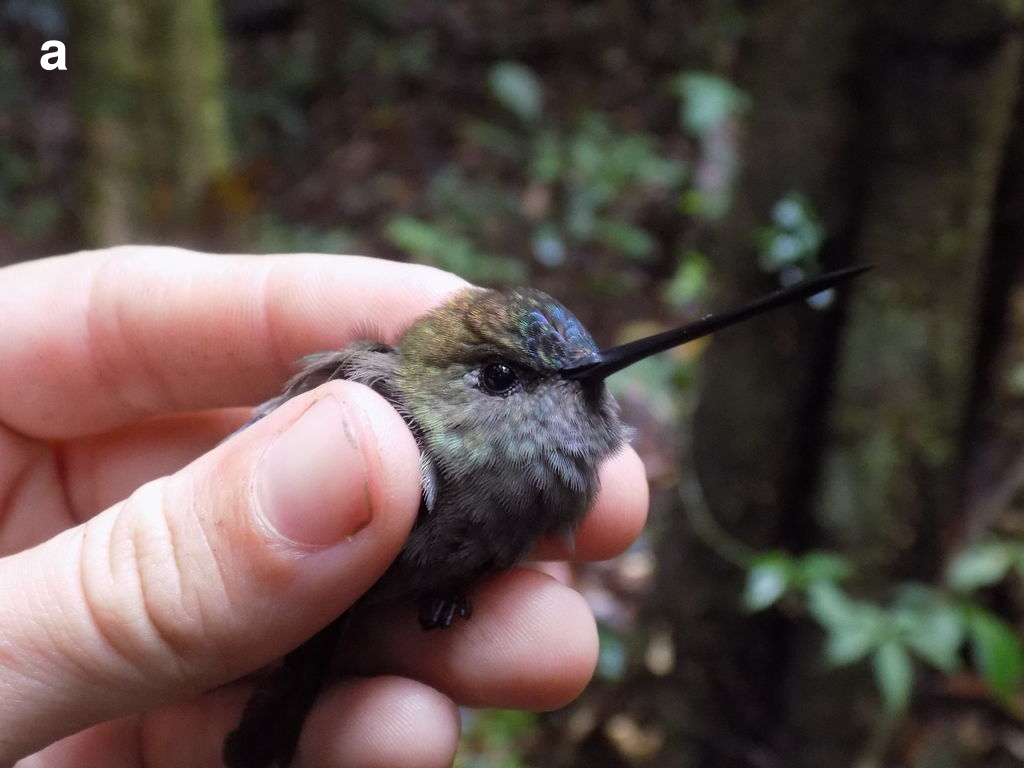
Blue-fronted Lancebill (*Doryfera johannae*)

**Figure 8b.**
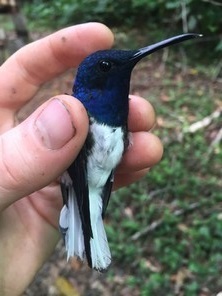
White-necked Jacobin (*Florisuga mellivora*)

**Table 1.** Putatively bird pollinated plants along the Manú Gradient.

Plant Genus	Specific epithet	Family	Collection number(s) for plant specimens deposited in the herbaria of MOL and UBC	Corolla length (mm)	Corolla width (mm)	Nectar (% sugar, * = not recorded)	Corolla colour(s)	Hummingbird visited	Habitat notes
*Besleria* L.	sp. 1	Gesneriaceae	MMAB 1	8	1	*	red		Growing along trail's edge in relatively open canopy
*Heliconia* L.	sp. 1	Heliconiaceae	MMAB 10, 11	100	19	*	translucent with pink		Lowest point of a bog with little shade. Pioneering Cecropia and Schefflera are dominating species. Ruellia also abundant.
* Centropogon *	*granulosus* C. Presl	Campanulaceae	MMAB 12, 13	40	15	*	yellow within red bract		Along ditches of the Manu Road. Typically at points facing South-East. Relatively dry forest edge.
* Centropogon *	*granulosus* C. Presl	Campanulaceae	MMAB 2, 3	35	15	25.5	red and yellow		Vine growing through dense understory at 1-3 m. Guaduais abundant. Flowers at breaks in the canopy where sunlight is more abundant.
*Sanchezia* Ruiz & Pav.	sp. 1	Acanthaceae	MMAB 20, 21	70	5	*	yellow within red bract		Along ditches of the Manu Road. Grows along weedy species including Vernonia, Calceolaria, and Gloxinia
* Columnea *	*guttata* Poepp. & Endl.	Gesneriaceae	MMAB 22, 23	10	1	20	yellow		Epiphytic. Found readily in the same habitats as that of Columnea sp. 1
* Heliconia *	*subulata* Ruiz & Pav.	Heliconiaceae	MMAB 24, 44	40	10	*	bright yellow in dark red bract		Found in dense stand of Guadua. Little sunlight, relatively dry.
* Guzmania *	*weberbaueri* Mez	Bromeliaceae	MMAB 25, 26	37	7	*	yellow	*Colibri thalassinus*, *Doryfera ludovicae*, *Heliodoxa leadbeateri*	Grows both as an epiphyte and from the ground. Always in high-moisture environments including bogs but less common near river's edge.
* Columnea *	cf. inaequilatera Poepp. & Endl.	Gesneriaceae	MMAB 27, 28	44	9	21.5	red		Edge of fast-flowing rocky river with little shade.
*Thyrsacanthus* Moric	sp. 1	Acanthaceae	MMAB 29, 30	10	1	22.5	red-purple		Along ditch of the Manu Road. East-facing, well drained.
* Gurania *	*eriantha* Poepp. & Endl.	Cucurbitaceae	MMAB 33	*	*	*	red		Along ditch of the Manu Road. Growing through dense vegetation, flowers at edge.
* Drymonia *	*semicordata* (Poepp.) Wiehler	Gesneriaceae	MMAB 34, 35	10	2	*	yellow within red bract		Hanging over edge of fast-flowing rocky river. Shaded by various Araceae.
*Besleria* L.	sp. 1	Gesneriaceae	MMAB 36, 37	20	8	*	bright red		Wet, dark, steep rocky cliff. North facing.
* Drymonia *	*urceolata* Wiehler	Gesneriaceae	MMAB 40, 39	20	5	*	red		Wet, dark, steep rocky cliff. North facing.
*Erythrina* L.	sp. 1	Fabaceae	MMAB 42, 41	*	*	*	orange-red		Flowers found on ground at the lowest point of a bog with little shade.
* Passiflora *	*coccinea* Aubl.	Passifloraceae	MMAB 43, 38	120	60	22.5	red	Unidentified	Growing from 0-12 m through dense stand of Guadua.
* Centropogon *	*congestus* Gleason	Campanulaceae	MMAB 45	32	10	*	pink-red		Dense stand of Guadua. Relatively humid and little light.
* Oreocallis *	*grandiflora* (Lam.) R. Br.	Proteaceae	MMAB 49, 48	46	12	*	red-purple	*Aglaeactis cupripenni*, *Boissonneaua matthewsii*	Dominating tree species in dry, scrubby, elfin forest.
* Siphocampylus *	*scandens* (Kunth) G.Don	Campanulaceae	MMAB 50, 51	47	8	16	pink	*Adelomyia melanogenys*	Along ditch of the Manu Road. Grows indiscriminately in sun or shade.
* Siphocampylus *	*orbignianus* A.DC.	Campanulaceae	MMAB 52, 53	54	17	12	pink-red	*Coeligena* sp.	Along ditch of the Manu Road. Grows indiscriminately in sun or shade.
* Brachyotum *	*rostratum* (Naudin) Triana	Melastomataceae	MMAB 54, 55	19	7	13	red with yellow tip	*Aglaeactis cupripenni*	Dry scrubby elfin forest. Dead ferns make up dense mat up to 1 m.
* Aetanthus *	*nodosus* (Desr.) Engl.	Loranthaceae	MMAB 56, 57	70	5	14.5	dark purple	*Coeligena* sp.	Humid transitional forest at where elfin forest dimishes.
*Gaultheria* Kalm *ex* L.	sp. 1	Ericaceae	MMAB 58, 59	7	3	*	red with yellow tip	*Aglaeactis cupripenni*, *Metallura tyrianthina*	Edge of pond alongside other Ericaceae species. Abundant light, south facing.
*Miconia* Ruiz & Pavón	sp. 1	Melastomataceae	MMAB 6, 7	15	13	*	pink	*Heliodoxa leadbeateri*	3 m tree mostly shaded by Cecropia and other taller species.
* Passiflora *	*mixta* L.f.	Passifloraceae	MMAB 60, 61	118	45	*	white-pink	*Ensifera ensifera*	Growing through same habitat as Ericaceae gen. sp. 1 and 2. Flowers at breaks in the canopy.
*Fuchsia* L.	sp. 1	Onagraceae	MMAB 63, 62	65	45	*	bright pink		Humid, dark understory. Habitat tends to be rocky.
*Desmodium* Desv.	sp. 1	Fabaceae	MMAB 64, 65	20	19	16.5	light red-orange	*Metallura* sp.	Rocky exposed cliffside. Many ferns. Dry.
*Siphonandra* Klotzsch	sp. 1	Ericaceae	MMAB 66, 67	18	5	*	purple	*Aglaeactis cupripenni*	Edge of pond alongside other Ericaceae species. Abundant light, south facing.
*Bomarea* Mirb.	sp. 1	Alstroemeriaceae	MMAB 68, 69	18	7	18.5	red with white tip		Rocky cliff next to slow-flowing river. In dense vegetation including Rubus and Asteraceae spp.
* Drymonia *	*semicordata* (Poepp.) Wiehler	Gesneriaceae	MMAB 70, 71	40	17	*	yellow within red bract	*Glaucis hirsutus*, *Heliodoxa aurescens*, *Phaethornis* sp.	Ubiquitous throughout humid lowland forest.
*Pachystachys* Nees	sp. 1	Acanthaceae	MMAB 72	60	8	*	red		In the shade of tall trees at trail's edge.
*Costus* L.	sp. 3	Costaceae	MMAB 75, 74	30	5	*	yellow		Relatively common at trail's edge, even in low light.
* Heliconia *	*densiflora* Verl.	Heliconiaceae	MMAB 76, 77	47	7	*	orange within red bract		High moisture depression in humid forest. Medium shade.
*Costus* L.	sp. 2	Costaceae	MMAB 78, 79	39	7	*	yellow within red bract		Terra firma approx 300 m from Rio Madre de Dios
* Columnea *	aff. schimpfii Mansf.	Gesneriaceae	MMAB 8, 9	30	5	*	white		Epiphytic. Can be found indiscriminately on any trees from at least 1-8 m.
*Besleria* L.	sp. 4	Gesneriaceae	MMAB 80, 81	22	8	*	red		Terra firma approx 300 m from Rio Madre de Dios
* Heliconia *	*schumanniana* Loes.	Heliconiaceae	MMAB 82, 83	44	5	*	yellow within red bract		Abundant sunlight at clearning in forest.
* Heliconia *	*lingulata* Ruiz & Pav.	Heliconiaceae	MMAB 84, 85	37	4	*	yellow within yellow bract		South-facing clay bank of the Alto Madre de Dios.
*Besleria* L.	sp. 2	Gesneriaceae	MMAB 86, 87	19	9	*	orange	Unidentified	Terra firma approx 300 m from Rio Madre de Dios
*Besleria* L.	sp. 3	Gesneriaceae	MMAB 88, 89	15	4	*	orange	Unidentified	Relatively exposed at trail's edge. Dense cluster of upto 20 individuals.
* Heliconia *	*metallica* Planch. & Linden ex Hook.	Heliconiaceae	MMAB 90, 91	40	4	*	red	*Phaethornis* sp.	High moisture depression in humid forest. Medium shade.
*Pentagonia* Benth.	sp. 1	Rubiaceae	MMAB 93, 92	31	10	*	yellow within red bract	Unidentified	High moisture depression in humid forest. Medium shade.
*Passiflora* L.	sp. 1	Passifloraceae	MMAB 94, 95	80mm long, pre-anthesis	*	*	red		Growing through dense understory including Melastomaceae.
*Pachystachys* Nees	sp. 2	Acanthaceae	MMAB 96, 97	50	17	*	red	*Phaethornis* sp.	Relatively exposed at trail's edge.

**Table 2.** Records of hummingbird-plant visitation along the Manú Gradient.

Hummingbird Species	Plant visited	Plant Family	Collection number	Site
*Adelomyia melanogenys* Bonaparte	*Siphocampylus scandens*	Campanulaceae	MMAB 50	San Pedro
*Aglaeactis cupripennis* Bourcier	*Gaultheria* sp. 1	Ericaceae	MMAB 58	Wayqecha
*Aglaeactis cupripennis*	*Siphonandra* sp. 1	Ericaceae	MMAB 66	Wayqecha
*Aglaeactis cupripennis*	*Brachyotum rostratum*	Melastomataceae	MMAB 54	Wayqecha
*Aglaeactis cupripennis*	*Oreocallis grandiflora*	Proteaceae	MMAB 49	Wayqecha
*Boissonneaua matthewsii* Bourcier	*Oreocallis grandiflora*	Proteaceae	MMAB 49	Wayqecha
*Coeligena* sp.	*Siphocampylus orbignianus*	Campanulaceae	MMAB 52	Wayqecha
*Coeligena* sp.	*Aetanthus nodosus*	Loranthaceae	MMAB 56	Wayqecha
*Colibri thalassinus* Swainson	*Guzmania weberbaueri*	Bromeliaceae	MMAB 25	San Pedro
*Doryfera ludovicae* Bourcier & Mulsant	*Guzmania weberbaueri*	Bromeliaceae	MMAB 25	San Pedro
*Ensifera ensifera* Lesson	*Passiflora mixta*	Passifloraceae	MMAB 60	Wayqecha
*Glaucis hirsutus* Gmelin	*Drymonia semicordata*	Gesneriaceae	MMAB 70	Pantiacolla
*Heliodoxa aurescens* Gould	*Drymonia semicordata*	Gesneriaceae	MMAB 70	Pantiacolla
*Heliodoxa leadbeateri* Bourcier	*Miconia* sp.1	Melastomataceae	MMAB 6	San Pedro
*Heliodoxa leadbeateri*	*Guzmania weberbaueri*	Bromeliaceae	MMAB 25	San Pedro
*Metallura tyrianthina* Loddiges	*Brachyotum rostratum*	Melastomataceae	MMAB 54	Wayqecha
*Metallura* sp.	*Desmodium* sp. 1	Fabaceae	MMAB 64	Wayqecha
*Metallura tyrianthina*	*Gaultheria* sp. 1	Ericaceae	MMAB 58	Wayqecha
*Phaethornis* sp.	*Pachystachys* sp. 2	Acanthaceae	MMAB 96	Pantiacolla
*Phaethornis* sp.	*Drymonia semicordata*	Gesneriaceae	MMAB 70	Pantiacolla
*Phaethornis* sp.	*Heliconia metallica*	Heliconiaceae	MMAB 90	Pantiacolla
Unidentified Trochilidae	*Besleria* sp. 2	Gesneriaceae	MMAB 86	Pantiacolla
Unidentified Trochilidae	*Besleria* sp. 3	Gesneriaceae	MMAB 88	Pantiacolla
Unidentified Trochilidae	*Passiflora coccinea*	Passifloraceae	MMAB 43	San Pedro
Unidentified Trochilidae	*Pentagonia* sp. 1	Rubiaceae	MMAB 93	Pantiacolla

**Table 3.** Basic bill morphometrics from birds mist-netted along the Manú Gradient.

Species	Sex (F=Female, M=Male, U=Unknown)	Mean bill length (mm)	Bill length std dev (mm)	Mean bill width (mm)	Bill width std dev (mm)	Bill length sample size	Bill width sample size
*Adelomyia melanogenys*	F	14.55	1.34	2.7	0	2	2
*Adelomyia melanogenys*	U	14.56	1.10	2.44	0.18	9	9
*Aglaeactis cupripennis*	U	18.06	1.05	2.64	0.11	5	5
*Boissonneaua matthewsii* Bourcier	U	18.4	NA	2.9	NA	1	1
*Chalcostigma ruficeps* Gould	F	11.5	NA	2	NA	1	1
*Chalcostigma ruficeps*	M	11.9	1.27	2.2	0.14	2	2
*Chlorostilbon mellisugus* Linnaeus	F	20.5	NA	2.7	NA	1	1
*Coeligena coeligena* Lesson	U	29.47	3.30	2.65	0.21	9	10
*Coeligena torquata* Boissonneau	M	32.4	1.9	2.66	0.20	3	3
*Coeligena torquata*	F	36.2	NA	3	NA	1	1
*Coeligena violifer* Gould	U	31.74	4.78	3.22	0.25	5	5
*Coeligena violifer*	M	33.13	0.98	3.26	0.25	3	3
*Coeligena violifer*	F	35.5	1.4	3.3	0.35	4	4
*Colibri coruscans* Gould	U	24.05	2.89	3.06	0.15	2	3
*Colibri thalassinus*	U	21.46	1.72	3	0.17	3	3
*Doryfera johannae* Bourcier	F	26.2	NA	3	NA	1	1
*Doryfera ludovicae*	U	27.62	7.90	2.75	0.14	7	8
*Doryfera ludovicae*	M	30.8	NA	2.6	NA	1	1
*Doryfera ludovicae*	F	31.2	1	2.5	0.26	3	3
*Eutoxeres condamini* Bourcier	U	24.23	1.52	3.87	0.68	8	7
*Florisuga mellivora* Linnaeus	F	18.1	NA	2.6	NA	1	1
*Florisuga mellivora*	M	18.7	0.28	3.45	0.49	2	2
*Glaucis hirsutus*	M	28.9	NA	3.3	NA	1	1
*Glaucis hirsutus*	U	29.15	1.21	3.82	0.29	4	4
*Heliangelus amethysticollis* d'Orbigny & Lafresnaye	M	17.46	0.69	2.46	0.29	6	6
*Heliangelus amethysticollis*	U	18.5	NA	2.5	NA	1	1
*Heliangelus amethysticollis*	F	18.65	0.21	2.8	0.14	2	2
*Heliodoxa leadbeateri*	M	20.56	0.99	2.95	0.05	6	6
*Heliodoxa leadbeateri*	U	20.8	NA	3.2	NA	1	1
*Heliodoxa leadbeateri*	F	22.27	0.82	3.2	0.16	4	4
*Ocreatus underwoodii* Lesson	F	15.6	NA	2.5	NA	1	1
*Ocreatus underwoodii*	M	15.8	NA	2.2	0.84	1	2
*Ocreatus underwoodii*	U	15.8	NA	NA	NA	1	0
*Phaethornis guy* Lesson	F	38.05	1.90	3.3	0.42	2	2
*Phaethornis guy*	U	39.6	NA	3	NA	1	1
*Phaethornis koepckeae* Weske & Terborgh	U	34.5	2.09	3.725	0.17	4	4
*Phaethornis superciliosus* Linnaeus	U	35.43	2.19	3.65	0.58	6	6
*Thalurania furcata* Gmelin	F	20.7	0.55	3.2	0.45	3	3
*Thalurania furcata*	M	23.65	4.03	3.55	0.49	2	2
*Thalurania furcata*	U	24.6	6.22	3.25	0.21	2	2
*Threnetes leucurus*	U	28.52	0.99	3.675	0.22	4	4

**Table 4.** Site information for putatively bird pollinated plants along the Manú Gradient

Collection numbers	Site	Latitude	Longitude	Altitude (m a.s.l.)	Date
MMAB 1	San Pedro	-13.056864	-71.546146	1347	4-ix-2016
MMAB 2, 3	San Pedro	-13.057179	-71.546566	1402	4-ix-2016
MMAB 6, 7	San Pedro	-13.057697	-71.547385	1393	4-ix-2016
MMAB 8, 9	San Pedro	-13.057311	-71.547086	1411	4-ix-2016
MMAB 10, 11	San Pedro	-13.058199	-71.547978	1403	4-ix-2016
MMAB 12, 13	San Pedro	-13.057907	-71.548086	1357	4-ix-2016
MMAB 20, 21	San Pedro	-13.054945	-71.545872	1378	6-ix-2016
MMAB 22, 23	San Pedro	-13.056268	-71.546039	1394	6-ix-2016
MMAB 24, 44	San Pedro	-13.05637	-71.54609	1355	7-ix-2016
MMAB 25, 26	San Pedro	-13.058848	-71.547884	1330	7-ix-2016
MMAB 27, 28	San Pedro	-13.059836	-71.54739	1360	7-ix-2016
MMAB 29, 30	San Pedro	-13.058044	-71.549996	1269	8-ix-2016
MMAB 33	San Pedro	-13.05773	-71.548458	1439	8-ix-2016
MMAB 34, 35	San Pedro	-13.057514	-71.543293	1324	8-ix-2016
MMAB 36, 37	San Pedro	-13.05634	-71.541812	1547	9-ix-2016
MMAB 40, 39	San Pedro	-13.054006	-71.539007	1297	9-ix-2016
MMAB 43, 38	San Pedro	-13.058459	-71.548074	1363	10-ix-2016
MMAB 42, 41	San Pedro	-13.058199	-71.547978	1403	11-ix-2016
MMAB 45	San Pedro	-13.191861	-71.588599	1149	16-ix-2016
MMAB 49, 48	Wayqecha	-13.173428	-71.587187	2727	20-ix-2016
MMAB 50, 51	Wayqecha	-13.17706	-71.586071	2939	21-ix-2016
MMAB 52, 53	Wayqecha	-13.179536	-71.585172	2958	21-ix-2016
MMAB 54, 55	Wayqecha	-13.180133	-71.585235	2955	22-ix-2016
MMAB 56, 57	Wayqecha	-13.174448	-71.587465	2888	26-ix-2016
MMAB 58, 59	Wayqecha	-13.176716	-71.581308	2625	26-ix-2016
MMAB 60, 61	Wayqecha	-13.174771	-71.588345	2866	27-ix-2016
MMAB 63, 62	Wayqecha	-13.174751	-71.588335	2904	27-ix-2016
MMAB 64, 65	Wayqecha	-13.191716	-71.586709	2834	28-ix-2016
MMAB 66, 67	Wayqecha	-13.18732	-71.585754	2979	28-ix-2016
MMAB 68, 69	Wayqecha	-13.173166	-71.591911	2780	29-ix-2016
MMAB 70, 71	Pantiacolla	-12.656352	-71.230691	398	6-x-2016
MMAB 72	Pantiacolla	-12.655418	-71.229373	391	7-x-2016
MMAB 75, 74	Pantiacolla	-12.656351	-71.230732	396	8-x-2016
MMAB 76, 77	Pantiacolla	-12.64719	-71.240662	394	8-x-2016
MMAB 78, 79	Pantiacolla	-12.645874	-71.234135	410	9-x-2016
MMAB 80, 81	Pantiacolla	-12.65622	-71.231045	404	9-x-2016
MMAB 82, 83	Pantiacolla	-12.656216	-71.230678	404	9-x-2016
MMAB 84, 85	Pantiacolla	-12.656545	-71.231864	405	11-x-2016
MMAB 86, 87	Pantiacolla	-12.656431	-71.231836	396	11-x-2016
MMAB 88, 89	Pantiacolla	-12.650034	-71.225302	428	12-x-2016
MMAB 90, 91	Pantiacolla	-12.651347	-71.22389	391	12-x-2016
MMAB 93, 92	Pantiacolla	-12.65138	-71.223853	397	12-x-2016
MMAB 94, 95	Pantiacolla	-12.651421	-71.223706	423	13-x-2016
MMAB 96, 97	Pantiacolla	-12.651113	-71.223842	394	13-x-2016

**Table 5.** Summary information and site descriptions for three sampling points along the Manú Gradient.

Site (Latitude, Longitude)	Period Collected and Netted	Altitudinal Range Sampled (m asl)	Number of Plants Species Collected	Number of Hummingbirds Netted	Number of Hummingbird Species Netted	Number of Bird Visits Recorded	General Site Description
San Pedro (-13.055387, -71.546832)	4-ix-2016 to 16-ix-2016	1149 - 1547	19	76	14	7	Montane cloud forest, *Cecropia* readily found in disturbed habitats. Dominant palm is Wettinia and canopy is generally composed of Clusiaceae, Rubiaceae, Melastomataceae and Lauraceae (Weng et al. 2004).
Wayqecha (-13.1752615, -71.5884099)	20-ix-2016 to 03-x-2016	2625 - 2979	11	65	15	10	Highland cloud forest and puna grassland of mainly Asteraceae and Poaceae. *Oreocalis grandiflora* is a noteable and abundant tree species. Araliaceae, Cunoniaceae,Chloranthaceae, Myrsinaceae, Sabiaceae, and Symplocaceae are readily found (Weng et al. 2004).
Pantiacolla (-12.656544, -71.231862)	07-x-2016 to 13-x-2016	391 - 428	14	31	9	8	Lowland rainforest, includes both seasonally flooded and terra firme forests. Canopy dominated by Fabaceae, Malvaceae, Moraceae and Annonaceae (Weng et al. 2004, Weng et al. 2004)
